# Transcriptomic and proteomic responses to very low CO_2_ suggest multiple carbon concentrating mechanisms in *Nannochloropsis oceanica*

**DOI:** 10.1186/s13068-019-1506-8

**Published:** 2019-06-28

**Authors:** Li Wei, Mohamed El Hajjami, Chen Shen, Wuxin You, Yandu Lu, Jing Li, Xiaoyan Jing, Qiang Hu, Wenxu Zhou, Ansgar Poetsch, Jian Xu

**Affiliations:** 1 Single-Cell Center, CAS Key Laboratory of Biofuels and Shandong Key Laboratory of Energy Genetics, Qingdao Institute of BioEnergy and Bioprocess Technology, Chinese Academy of Sciences, Qingdao, Shandong China; 2 Department of Plant Biochemistry, Ruhr University Bochum, Bochum, Germany; 3 Institute of Hydrobiology, Chinese Academy of Sciences, Wuhan, Hubei China; 4 Department of Chemistry and Biochemistry, Center for Chemical Biology, Texas Tech University, Lubbock, TX USA; 5 University of Chinese Academy of Science, Beijing, China; 6 School of Biomedical and Healthcare Sciences, University of Plymouth, Plymouth, UK

**Keywords:** Carbon fixation, Carbon concentrating mechanism (CCM), C4-like cycle, Industrial oleaginous microalgae, *Nannochloropsis oceanica*

## Abstract

**Background:**

In industrial oleaginous microalgae such as *Nannochloropsis* spp., the key components of the carbon concentration mechanism (CCM) machineries are poorly defined, and how they are mobilized to facilitate cellular utilization of inorganic carbon remains elusive.

**Results:**

For *Nannochloropsis oceanica*, to unravel genes specifically induced by CO_2_ depletion which are thus potentially underpinning its CCMs, transcriptome, proteome and metabolome profiles were tracked over 0 h, 3 h, 6 h, 12 h and 24 h during cellular response from high CO_2_ level (HC; 50,000 ppm) to very low CO_2_ (VLC; 100 ppm). The activity of a biophysical CCM is evidenced based on induction of transcripts encoding a bicarbonate transporter and two carbonic anhydrases under VLC. Moreover, the presence of a potential biochemical CCM is supported by the upregulation of a number of key C4-like pathway enzymes in both protein abundance and enzymatic activity under VLC, consistent with a mitochondria-implicated C4-based CCM. Furthermore, a basal CCM underpinned by VLC-induced upregulation of photorespiration and downregulation of ornithine–citrulline shuttle and the ornithine urea cycles is likely present, which may be responsible for efficient recycling of mitochondrial CO_2_ for chloroplastic carbon fixation.

**Conclusions:**

*Nannochloropsis oceanica* appears to mobilize a comprehensive set of CCMs in response to very low CO_2_. Its genes induced by the stress are quite distinct from those of *Chlamydomonas reinhardtii* and *Phaeodactylum tricornutum*, suggesting tightly regulated yet rather unique CCMs. These findings can serve the first step toward rational engineering of the CCMs for enhanced carbon fixation and biomass productivity in industrial microalgae.

**Electronic supplementary material:**

The online version of this article (10.1186/s13068-019-1506-8) contains supplementary material, which is available to authorized users.

## Introduction

Elevated atmospheric CO_2_ levels, one consequence of fossil fuel use and deforestation, are leading to global warming and oceanic acidification. In nature, ~ 40% of the atmospheric CO_2_ was consumed by marine microalgae via photosynthesis [1–3], largely due to their enormous biomass, wide ecological distribution and high carbon fixing rates [4]. Such carbon sequestration capability is being exploited for industrial production of clean fuels and materials via cultivation of industrial microalgae, as many of them are able to convert sunlight and CO_2_ into energy-dense macromolecules (e.g., triacylglycerol; TAG) as well as high-value products (e.g., eicosapentaenoic acid). However, the inadequate productivity of microalgal biomass and oil, at both ambient (air level; 0.04% v/v) and elevated (flue gas level; usually > 5% v/v) CO_2_ concentrations [5, 6], has severely hindered the efforts to fulfill these promises [7].

To tackle this challenge, mechanistic insights into substrate intake machineries of these industrial oleaginous microalgae, in particular carbon concentrating mechanisms (CCMs; [8]), are essential. As a feat of plant evolution driven by gradual reduction in atmosphere CO_2_ concentration (down to the 0.04% v/v at present), CCMs act as a dissolved inorganic carbon (DIC) pump to increase CO_2_ concentration in the vicinity of RuBisCO [9]. So far, various types of CCMs have been discovered in plants and algae. Generally, there are mainly three types of CCMs including C3, C4 and CAM (crassulacean acid metabolism) in plants [10], while biophysical and/or biochemical CCMs are employed in microalgae [11]. The C3 pathway is present in plants and algae, which is known as the Calvin cycle by producing a three-carbon compound called 3-phosphoglyceric acid. The C4 carbon fixation was found in higher plants (maize, sorghum and sugarcane) and in diatoms (*Thalassiosira weissflogii*), and CAM is present in cactus, pineapple and orchid [10]. As for microalgae, in the biophysical CCM, carbonic anhydrases (CAs) and bicarbonate transporters (BCTs) collaborate to interconvert between the inorganic carbon forms and transport them across the various membranes [12]. In the biochemical CCM (also called the C4-like pathway), HCO_3_^−^ is converted into oxaloacetate (a C4 compound) by phosphoenolpyruvate carboxylase (PEPC), which is then decarboxylated into CO_2_ and malate by malate dehydrogenase (MDH) and/or malic enzyme (ME); the CO_2_ then enters the Calvin cycle [9, 11, 13]. In the basal CCM, mitochondrial γ-type CAs and NADH–ubiquinone oxidoreductase complex I of the respiratory chain recycle mitochondrial CO_2_ for the carbon fixation in chloroplasts and thus reduce the leakage of CO_2_ from plant cells (e.g., *Arabidopsis*; [14]). Despite these important roles, activities of these CCMs are all highly regulated in the cell. For example, in eukaryotic microalgae such as *Chlamydomonas reinhardtii* and diatoms, biochemical and biophysical CCMs are quite sensitive to extracellular carbon level and are activated below air-level CO_2_ [9, 15], although the response of basal CCM to carbon level is less well defined.

The diverse CCMs are not universally found in all microalgae, and even species from a genus can employ distinct CCMs [15, 16]. Among the three kinds of CCMs, the biophysical CCM including its key components of CAs and BCTs is the most widely distributed [9]. For the biochemical CCM, although the C4-like pathway genes are widely present in microalgal genomes, not all microalgae that harbor these genes actually employ them for carbon concentrating purposes. For instance, the C3 carbon fixation (conversion of CO_2_ and ribulose bisphosphate into two molecules of 3-phosphoglycerate, which occurs in all plants as the first step of the Calvin–Benson cycle), but not the biochemical CCM, is found in *C. reinhardtii* [17]. In the diatoms, although a C3 pathway is used by *Phaeodactylum tricornutum* [18], a C4-like carbon fixation pathway is employed by *Thalassiosira weissflogii*, and a C3–C4 intermediate carbon fixation pathway is present in *Thalassiosira pseudonana* [11, 16, 19]. As for basal CCM, its role in microalgae is poorly defined since it is not clear whether such systems present in higher plants such as *Arabidopsis thaliana* are employed in microalgae [14].

*Nannochloropsis* spp., a group of industrial oleaginous microalgae, have emerged as one research model for converting industrial sources of CO_2_ to oils, due to their rapid photosynthetic growth, high contents of TAG and eicosapentaenoic acids, tolerance to various environmental conditions and amenability to genetic manipulation [20–26]. Only recently, efforts to dissect and engineer *Nannochloropsis* CCMs have started, e.g., in *N. oceanica*, one α-type carbonic anhydrase (CA) named CAH1 that is localized to the lumen of the epiplastid endoplasmic reticulum provides a biochemical role in CCM function [27]; on the other hand, a cytosolic β-type CA called CA2 serves a pivotal role in maintaining the intracellular pH equilibrium upon elevated extracellular acidity, and its knockdown led to acidity-tolerant phenotypes [28]. Despite these discoveries, the global picture of the CCM machineries is poorly defined, and how the individual CCMs and their components are mobilized to facilitate cellular utilization of inorganic carbon remains elusive in this and related microalgae.

To address these questions, here for *Nannochloropsis oceanica* IMET1, we tracked the transcriptomic, proteomic and metabolomic profiles over 0 h, 3 h, 6 h, 12 h and 24 h during microalgal adaption from high CO_2_ level (HC; 50,000 ppm, or 5% v/v) to very low CO_2_ (VLC; 100 ppm, or 0.01%). The transcripts and proteins that are specifically induced by VLC indicate the coordinated activities of multiple CCMs including biophysical, biochemical and basal levels, which are mobilized in response to the very low level of CO_2_. This genome-wide, time-resolved choreography of transcriptome, metabolome and proteome, which is quite distinct from those of the laboratory model microalga *C. reinhardtii * and *Phaeodactylum tricornutum*, paves the way for mechanistically probing and rationally engineering individual nodes in the *N. oceanica* gene network for enhanced CO_2_ fixation and biomass production.

## Results and discussion

### Overview of *N. oceanica* physiological, transcriptomic, proteomic and metabolomic responses in VLC as compared to HC

To identify the molecular components of CCMs and delineate their interactions, the physiological, transcriptomic, proteomic and metabolomic responses were tracked under two contrasting culture conditions for *N. oceanica* (strain IMET1): VLC of 100 ppm and HC of 50,000 ppm (with HC being the reference: cells were cultured under HC before the split into VLC and HC; Additional file 1: Fig. S1; “Materials and methods”). Microalgal growth slowed down (~ 23% lower than HC) under VLC, yet remained vigorous under HC (Additional file 2: Fig. S2A). Under VLC, photosynthetic efficiency is merely ~ 15% lower than HC, as indicated by reduction in Fv′/Fm′ (active activity) and Fv/Fm (maximum activity) of Photosystem II (Additional file 2: Fig. S2B; “Materials and methods”), despite the ~ 62% to 300% lower dissolved inorganic carbon (DIC) in the medium (Additional file 2: Fig. S2C); this is consistent with the presence of a highly efficient CCM under VLC.

**Fig. 1:**
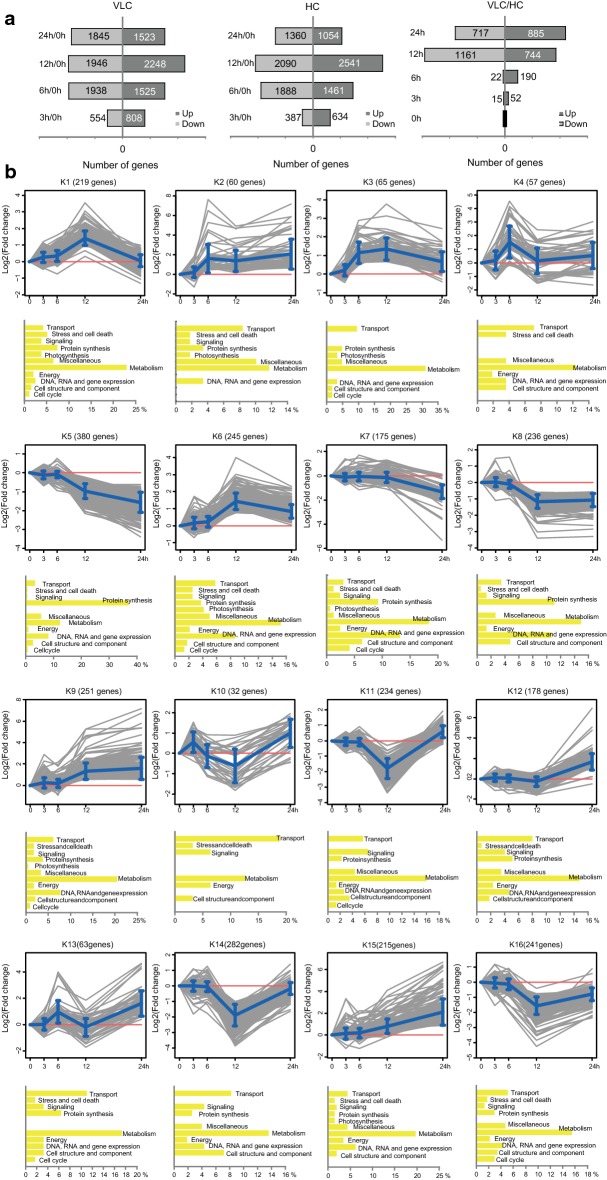
Transcriptome dynamics in the wild-type *N. oceanica* under VLC and HC. **a** Number of up- or downregulated genes at each of the time points under VLC (left; relative to 0 h), HC (middle; relative to 0 h) and VLC/HC (right). **b** Temporal patterns of relative transcript abundance (VLC/HC) for the 2933 differentially expressed genes are grouped into 16 clusters. Mean fold change [as computed by log_2_(VLC/HC)] of genes in a given cluster is plotted as light blue lines, with error bars showing standard deviation of the biological replicates. Manually annotated functional categories are shown below each cluster

Time series of transcriptomes were compared between VLC and HC by mRNA-Seq over the five time points of 0 h, 3 h, 6 h, 12 h and 24 h (Additional file 3: Table S1, Additional file 4: Table S2, Additional file 5: Table S3; Additional file 1: Fig. S1, Additional file 6: S3, Additional file 7: S4). For both VLC and HC, abundance of > 1000 genes altered at as early as 3 h, with > 2000 change at later hours, which underscores the global and profound impact of sampling time (Fig. 1a middle) and the VLC switch (Fig. 1a left) on transcriptome. Masking of time-dependent variations (i.e., VLC vs. HC) then reveals the cellular program of HC-to-VLC adaptation, which started with relatively few genes at early hours (67 and 212 genes for 3 h and 6 h, respectively; most downregulated) and then peaks at 12 h (1905 genes; Fig. 1a right). Such CO_2_ level-dependent changes are global and profound, as > 30% of genes were differentially transcribed during at least one time point after 0 h, with both upregulated and downregulated genes heavily represented [VLC versus HC using a cutoff value of one loget (i.e., log2-fold change); *p *< 0.05; Fig. 1a right; Additional file 8: Dataset S1].

Temporal patterns of these 2933 differentially regulated genes (i.e., VLC vs HC) over five time points formed 16 clusters (K1–K16; Fig. 1b). To inform whether a given pattern was linked to any specific functions, annotated genes in each cluster were manually categorized into twelve functional categories, with the largest category in each cluster (except for unknown genes) designated as its primary functional genes (Additional file 8: Dataset S1; Fig. 1b). In K1, the 12 h peak was functionally enriched with Calvin cycle and light-harvesting genes, which were upregulated at 12 h and then downregulated at 24 h. The primary functional genes of K2, K3 and K6 (these clusters show an upregulated trend at 6 h, 12 h and 24 h) included those involved in biophysical CCM, biochemical CCM, photorespiration and THF cycle. In K5, K7, K8 and K14 (showing a downregulated trend), genes related to DNA/RNA metabolism and gene expression (e.g., enzymes required for DNA replication and RNA transcriptional regulators) and those in protein synthesis and modification are enriched. In K14 and K16 (downregulated at 12 h and then rebounding at 24 h), TCA cycle genes were enriched. Many K5 genes encoded ribosomal proteins, which were downregulated by ca. twofold, indicating that the carbon flow, in response to VLC, might switch from protein synthesis to other pathways (e.g., gluconeogenesis and secondary metabolite biosynthesis).

**Fig. 2:**
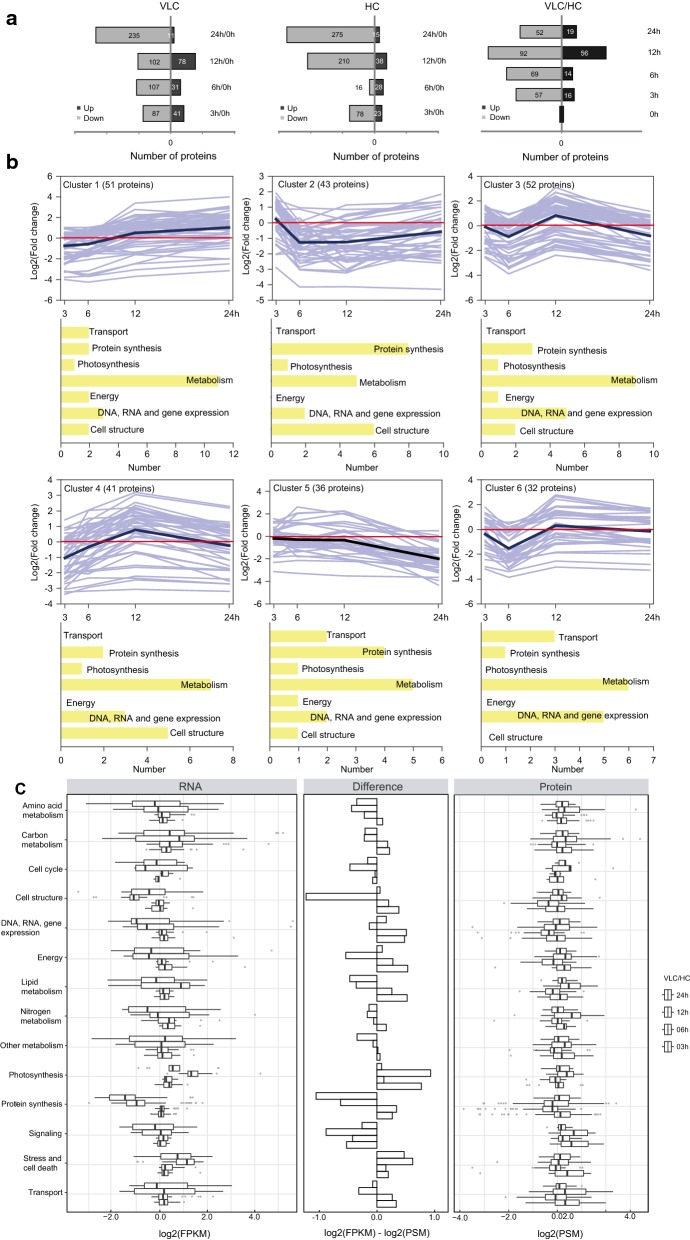
Proteome dynamics in the wild-type *N. oceanica* under VLC/HC. **a** Number of up- or downregulated proteins at each of the time points under VLC (left; relative to 0 h), HC (middle; relative to 0 h) and VLC/HC (right). **b** Temporal patterns of relative protein abundances at 3 h, 6 h, 12 h and 24 h. Clustering of *k*-means using Pearson’s correlation produced six clusters via similarity in abundance profiles. The *y*-axis represents the peptide–spectrum match (PSM). In a cluster, each blue line represents an individual protein, with the median pattern of abundance indicated by a dark blue line. For each cluster, functional categories of its proteins are shown. **c** Overview of gene expression values for mRNA-Seq (left), proteomics (right) and their difference (middle), with samples grouped at four time points from each culture condition. The data represent averages of expression values for genes (FPKM: fragments per kilobase million) and proteins (PSM: peptide–spectrum matches) assigned to selected functional categories

To track proteome dynamics, tryptic protein digests from samples matching the transcriptome series were measured by ESI LC–MS/MS on LTQ Orbitrap Elite (“Materials and methods”). High biological reproducibility was achieved among replicates (average Pearson correlation coefficient of 0.879). Altogether 3177 protein sequences (supplemental proteome files in PRIDE; PXD010030) were found (i.e., ~ 30% of the total 10,566 proteins encoded in the *N. oceanica* genome [20–22], with > 20% of them observed throughout the 24-h period). When considering only uniquely identified protein groups (i.e., proteins with same identified peptide sequences were grouped), a total of six clusters representing 1965 protein accessions were observed (Fig. 2a; Additional file 9: Dataset S2; “Materials and methods”). For both VLC and HC, > 100 proteins were differentially expressed (as compared to 0 h; > 77% upregulated) at 3 h and more at later hours (Fig. 2a middle and left). Comparing variations between the two cultivation approaches (i.e., VLC vs. HC) revealed that the regulation on proteome level increased over time and peaked at 12 h (72% of the changed proteins were upregulated; Fig. 2a right). The temporal patterns of regulated proteins formed six major clusters identified by the *k*-means algorithm in the MeV software package (Fig. 2b; “Materials and methods”). To inform whether a given pattern was linked to any specific functions, the protein groups in each cluster were manually categorized into seven functional categories, with the largest category in each cluster (ignoring unannotated proteins) designated as its primary functional proteins (Fig. 2b). In all six clusters, the biggest groups are proteins related to the metabolism or to the protein synthesis. In Cluster 1, the biggest group is related to metabolism (mostly components of CCMs, glycolysis/gluconeogenesis and TAG-lipid synthesis), which was first downregulated at 6 h and then upregulated at 12 h. Cluster 2 mainly consists of proteins related to nitrogen metabolism, which were slightly downregulated at 6 h and 12 h. In Cluster 3 the biggest group was protein synthesis, which was downregulated at 12 h and upregulated at 24 h; here, particularly enzymes of the amino acid degradation pathway were affected. In Clusters 4 and 5, proteins of metabolism were upregulated (Cluster 4 at 6 h and 12 h; Cluster 5 at 12 h and 24 h). Most important enzymes regulated in Cluster 4 were from CCM, TCA cycle, amino acid and the lipid pathway. In Cluster 5, mainly enzymes of lipid synthesis, carbon metabolism and CCM were affected. Cluster 6 showed downregulation (at 12 h and 24 h) of metabolism and protein synthesis-related protein groups, of these mostly lipid synthesis, CCM, citric acid cycle and glycolysis/gluconeogenesis.

For the various functional categories, temporal dynamics of transcriptome and proteome are mostly consistent, despite higher variance of the former in general (Fig. 2c). For example, in photosynthesis an upregulation of factor 1 (log2(FPKM)/log2(PSM)) on transcriptome at 12 h was apparent as compared to other time points, though not on proteome. The protein synthesis, modification, folding and turnover group showed a downregulation on transcriptome at 12 h and 24 h (factor 1 to 2), yet on proteome the downregulation is below a factor of 1 at 6 h and 12 h. In the lipid metabolism group, transcriptome and proteome both showed upregulation at 12 h, yet differ at the other time points. Nitrogen metabolism was downregulated on transcriptome for 12 h and 24 h, while upregulated on proteome at 12 h.

**Fig. 3:**
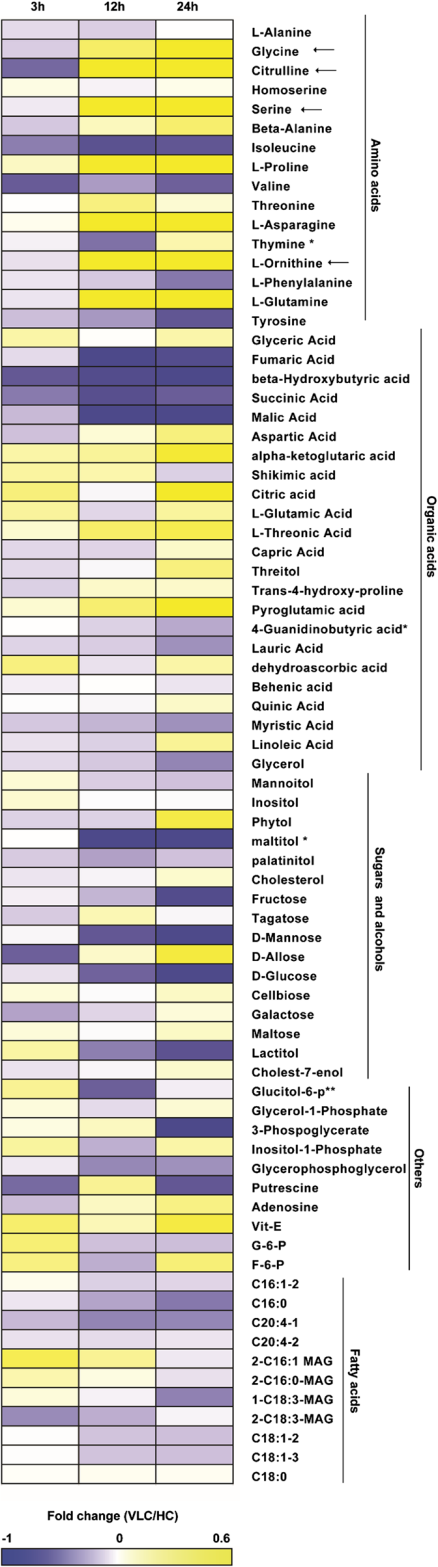
Metabolome dynamics in the wild-type *N. oceanica* under VLC/HC. Dynamics of selected metabolites under VLC/HC, where totally 18 samples were analyzed by GC–MS in triplicates at 3, 12 and 24 h under VLC and HC. The horziontal arrows represent those amino acids highlighted in Fig. 7B

Finally, to simultaneously track the metabolome profile, dynamics of over 100 polar and nonpolar compounds in central carbon metabolism and photorespiration were revealed via the same set of samples via GC–MS (“Materials and methods”). Relative abundances of 23 of them, mostly amino acids and organic acids, were altered between VLC and HC. The increased amino acids include glycine, citrulline, serine, alanine, proline, ornithine, glutamine and asparagine, while valine, isoleucine and tyrosine are decreased under VLC. However, sugars, polyols and nonpolar compounds were mostly unchanged (Fig. 3; Additional file 10: Dataset S3); among them, maltitol, lactitol, fructose and mannose were decreased under VLC. Additionally, biochemical compositions in carbohydrates, proteins and lipids were compared under VLC and HC. The content of proteins was higher under HC than that under VLC, while the contents of carbohydrates and lipids were essentially unchanged (Additional file 11: Fig. S5; “Materials and methods”). This suggests that protein synthesis slowed down under VLC, consistent with the downregulation of protein synthesis genes in both transcript and protein. Thus, majority of the profiled metabolites were not affected by the reduced CO_2_ level.

### Induction of bicarbonate transporter and two carbonic anhydrases under VLC suggests the presence of a biophysical CCM

**Fig. 4:**
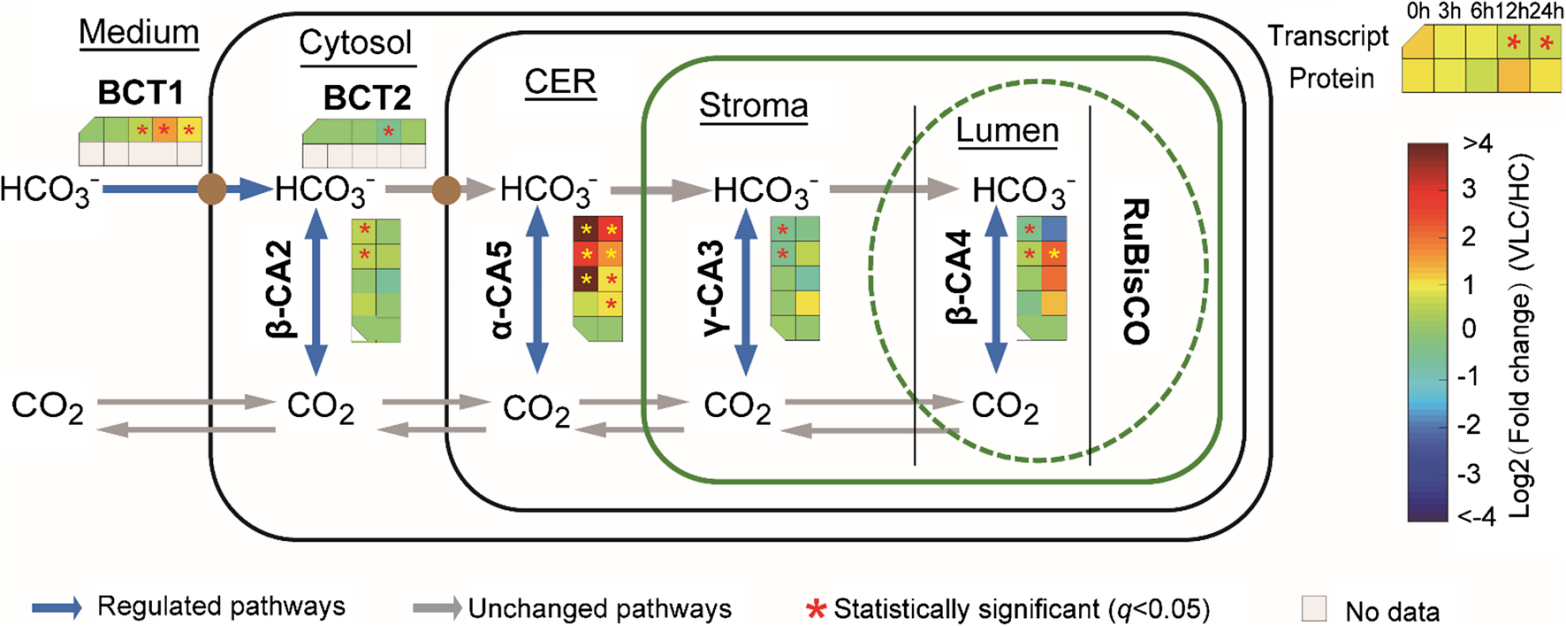
Change of the biophysical CCM pathway in *N. oceanica* under VLC/HC. Dynamics of transcripts and proteins are shown by heatmap at the various time points. Fold changes are calculated under VLC/HC at the time points by FPKM (for mRNA-Seq) values and peptide–spectrum matches (PSMs). CA, carbonic anhydrase; BCT, bicarbonate transporter; CER, chloroplast endoplasmic reticulum; PPC, periplastidal compartment

Carbonic anhydrases (CAs) play a key role in biophysical CCM, particularly under low CO_2_ level, as they are localized in cytosol and organelles (mitochondria or chloroplast, including chloroplast endoplasmic reticulum) and catalyze the interconversion between CO_2_ and HCO_3_^−^ [9]. In the *N. oceanica* IMET1 genome, four putative CAs (CA2: g2018; CA3: g2209; CA4: g4812; and CA5: g6125) could be linked to biophysical CCM [20, 21, 28] and predicted to target various cellular compartments (Fig. 4; *Nannochloropsis* spp. produce no extracellular CAs [29, 30], indicating a unique CCM where the cell possesses active bicarbonate transport systems).

Under VLC/HC, three of the CA transcripts (CA2, CA4, CA5) were upregulated (Fig. 4); such induction of specific CAs by low CO_2_ stress suggests an active biophysical CCM. Specifically, CA5 transcript (α-type; with type II signal peptide) showed a steady upregulation at 3 h, 6 h, 12 h and 24 h (0.7-, 4.3-, 2.7- and 4.9-loget, respectively) under VLC/HC (also upregulated at the protein level; Fig. 4); moreover, under VLC its transcripts increased by 1.6- and 2.5-loget at 6 h and 24 h as compared to 0 h (Additional file 8: Dataset S1). The active role of CA5 in carbon fixation was supported by the recent discovery of CAH1, an *N. oceanica* CCMP1779 homolog of CA5, as an essential, ER lumen-targeted component of CCM [27]. On the other hand, CA2 (β-type) g2018 steadily increased in abundance under VLC, suggesting its active role in adaptation to low CO_2_ (Additional file 8: Dataset S1; [28]). Interestingly, under nitrogen depletion (N−), g2018 was the only CA whose transcript was upregulated versus nitrogen replete condition (N+), by 0.9-, 1.2- and 1.5-loget at 12 h, 24 h and 48 h, respectively [21], indicating auxiliary regulation of the CCM by *N* availability.

*Nannochloropsis oceanica* genome harbors two bicarbonate transporters considered responsible for HCO_3_^−^ uptake in CCM: BCT1 (g19) and BCT2 (g1855). Both belong to the solute carrier protein 4 (SLC4) family and share 47% and 46% amino acid sequence identity with their known diatom counterpart (XP_002177487.1, which can directly pump HCO_3_^−^ from seawater for photosynthetic carbon fixation [31]). During CCM induction, g19 transcript was upregulated by over 1.0-loget under VLC/HC (Fig. 4), yet downregulated (0.6-loget) at the onset of N− (i.e., under N−/N+ [21]). In addition, transcripts of several ABC transporters (g6647, g6756) and SLC26 family proteins (g6134, g9142 and g9968), which might also be BCTs, were upregulated at 6 h and 24 h under VLC/HC. Thus, the supply of carbon to the Calvin cycle is likely not by passive CO_2_ diffusion but via coordination of such high-affinity uptake transporters for bicarbonate. Collectively these evidences support an active biophysical CCM under VLC.

### Induction of transcript abundance and enzymatic activity of C4-like genes under VLC indicates an active biochemical CCM

**Fig. 5:**
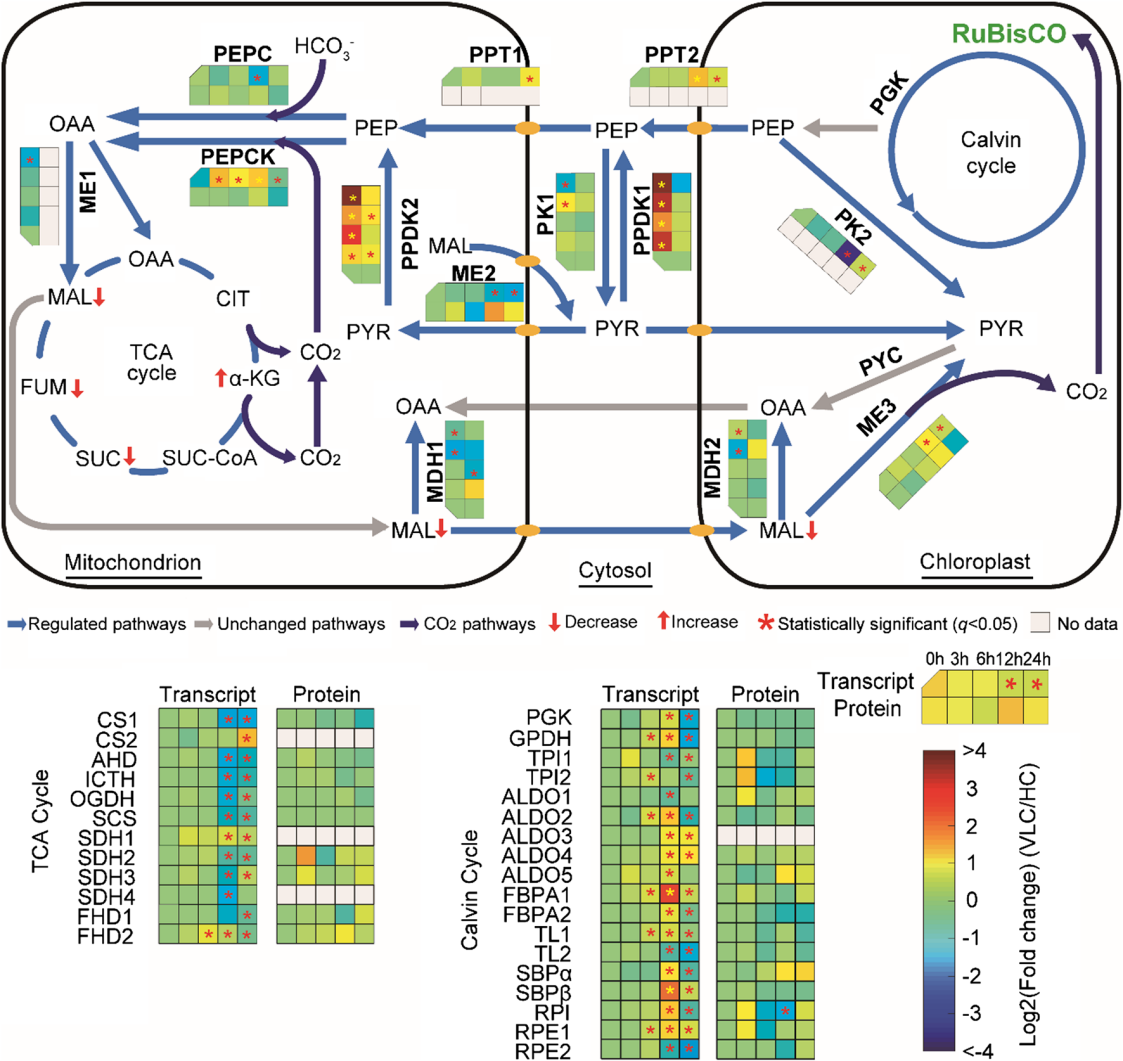
Change of the biochemical CCM pathway in *N. oceanica* under VLC/HC. PEPC, phosphoenolpyruvate carboxylase; PEPCK, phosphoenolpyruvate carboxylase kinase; PPDK, pyruvate orthophosphate dikinase; MDH, malate dehydrogenase; ME, malic enzyme; PK, pyruvate kinase; PYC, pyruvate carboxylase; PPT, Pi/PEP translocator; SFC, succinate/fumarate carrier; OAA, oxaloacetate; CIT, citrate; FUM, fumarate; SUC, succinate; α-KG, α-ketoglutarate; PEP, phosphoenolpyruvate; PYR, pyruvate; MAL, malate. The metabolites up- or downregulated based on the metabolomics analysis are indicated by red arrows

The biochemical CCM (e.g., in the diatom *Thalassiosira weissflogii*) involves the C4 pathway: fixing HCO_3_^−^ into C4 compounds (oxaloacetate) by phosphoenolpyruvate carboxylase (PEPC), decarboxylating oxaloacetate into CO_2_ and malate by malate dehydrogenase (MDH) and/or malic enzyme (ME), and then utilizing CO_2_ in the Calvin cycle [9, 11, 13]. These C4-like genes are present in *N. oceanica*, consistent with a potential C4 cycle (Fig. 5; Additional file 12: Table S4; Additional file 13: Fig. S6; “Materials and methods”). Intriguingly, PEPC that β-carboxylates phosphoenolpyruvate (PEP) to oxaloacetate (OAA) in the presence of HCO_3_^−^ and Mg^2+^ (or Mn^2+^) and phosphoenolpyruvate carboxylase kinase (PEPCK) which converts oxaloacetate (OAA) to phosphoenolpyruvate (PEP) are both predicted to target mitochondria in *N. oceanica* (Additional file 12: Table S4), although they are chloroplastic in higher plants [32]. In both *N. gaditana* and *N. oceanica*, HCO_3_^−^ uptake can persist in darkness for 20 min or longer [29, 33], indicating that supply of inorganic carbon for photosynthetic CO_2_ fixation might partially rely on mitochondria via the associated PEPC and PEPCK activities [34]. Thus, *N. oceanica* can potentially employ PEPC and PEPCK as the primary inorganic carbon fixation step in a C4-like pathway in mitochondria, followed by chloroplastic ME-mediated decarboxylation of malate to enrich CO_2_ in the chloroplast. Such a mitochondria-implicated C4-based CCM is not previously known to exist in microalgae.

**Fig. 6:**
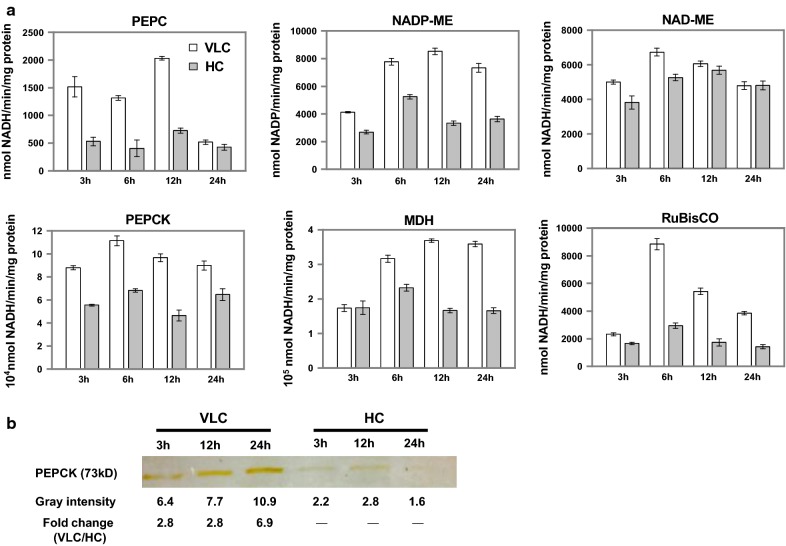
Enzymatic assays of key biochemical CCM genes in *N. oceanica* under VLC/HC. **a** Enzyme activities of C4-like genes under VLC and HC, including ME, MDH, PEPC and PEPCK. **b** Quantification of PEPCK protein under VLC and HC. Immunoblot band was detected from the cellular extract under VLC but not under HC (loading of 15 μg total protein)

For C4-like genes, although transcript abundance of PEPC, MDH and ME (including NAD-ME and NADP-ME) is largely insensitive to variation in CO_2_ level (Fig. 5), PEPC protein abundance shows a 0.3-loget upregulation at 3 h between VLC and HC proteomes (Fig. 5; Additional file 9: Dataset S2). Moreover, the enzymatic activities of PEPC, MDH and NADP-ME all exhibit an increase in 1–2-logets, as measured from the cellular extracts (elevation by ~ fourfold for PEPC; Fig. 6a; “Materials and methods”). Thus, the activity of these key C4-like enzymes might be regulated primarily at the protein level (e.g., allosteric and posttranslational regulation as in *Opuntia ficus*-*indica* [35]) and by pH or diurnal cycle [9]. Notably, the increase in PEPC activity might optimize the use of available phosphoenolpyruvate for carbon fixation, while saving pyruvate for acetyl-CoA formation. Consistently, for PEPCK (g6884), the transcript was upregulated up to 1.0-loget at 6 h under VLC (Fig. 5), whereas its enzyme activity was 1.0-loget higher (Fig. 6a). In fact, the PEPCK protein was detected by immunoblot under VLC but not under HC (Fig. 6b), which confirms the induction of PEPCK in both protein abundance and enzyme activity in response to the reduced level of CO_2_. Though PEPCK usually functions as OAA-decarboxylating enzyme, the reaction can favor OAA synthesis (e.g., in *Actinobacillus* [36] and *E. coli* [37]) as it is thermodynamically reversible. Thus, PEPCK working in the direction of OAA synthesis may occur under high PEP concentrations and low OAA concentrations in mitochondria. This hypothesis is underpinned by the VLC induction of other C4-like genes converting pyruvate into PEP, such as pyruvate orthophosphate dikinase (PPDK) and transcripts for the three PPDKs (g3407, g5453 and g5454), which were upregulated by 2.3–4.3-loget, respectively (Fig. 5). Notably, two of the PPDKs (g5453 and g5454) showed a 0.6-1.0-loget upregulation by proteome analysis (Fig. 5).

Changes in TCA metabolite pools under VLC were revealed by metabolomics. Although the level of α-ketoglutaric acid (α-KG) increased, those of succinate, fumarate and malate decreased (Fig. 5; Additional file 10: Dataset S3). This might be a consequence of higher malate consumption in the chloroplast under the presumption of malate shuttling carbon from mitochondrion to chloroplast. Increased anaplerotic OAA formation by PEPC and PEPCK would replenish TCA cycle under increased malate efflux. Furthermore, the unusual mitochondrial localization of PEPC and PEPCK might allow salvaging of CO_2_ released in the decarboxylation reactions of the TCA cycle. Taken together, the culminating evidences of transcriptomic, proteomic, metabolomic and enzyme activity data support the notion of a biochemical CCM under VLC in this organism.

### A plantlike basal CCM underpinned by photorespiration, the THF cycle and the ornithine urea cycle is suggested

A “basal” CCM that involves mitochondrial CAs has been proposed in higher plants, like *Arabidopsis* and maize, since the γ-type CAs in mitochondria are present in almost all photosynthetic eukaryotic organisms, but not in animals or fungi [14]. The γ-type CAs can attach to NADH–ubiquinone oxidoreductase complex I (CI) of the respiratory chain and form a spherical extra domain (named CA domain) on the matrix side of its membrane arm [14]. The CA domain (interacting with mitochondrial carbonic anhydrase) of CI forms part of a mitochondrial bicarbonate export system which enables the efficient transfer of mitochondria-produced CO_2_, in the form of bicarbonate and via mitochondrial decarboxylation reactions (including TCA cycle and photorespiration), into the chloroplast [38]. In Arabidopsis, those mutants defective in two mitochondrial CA subunits show an altered photorespiratory phenotype [39], which suggests that mitochondrial CA and CI are linked to photorespiration. This “basal” CCM can account for 10–20% of carbon fixation in C3 plants [14].

**Fig. 7:**
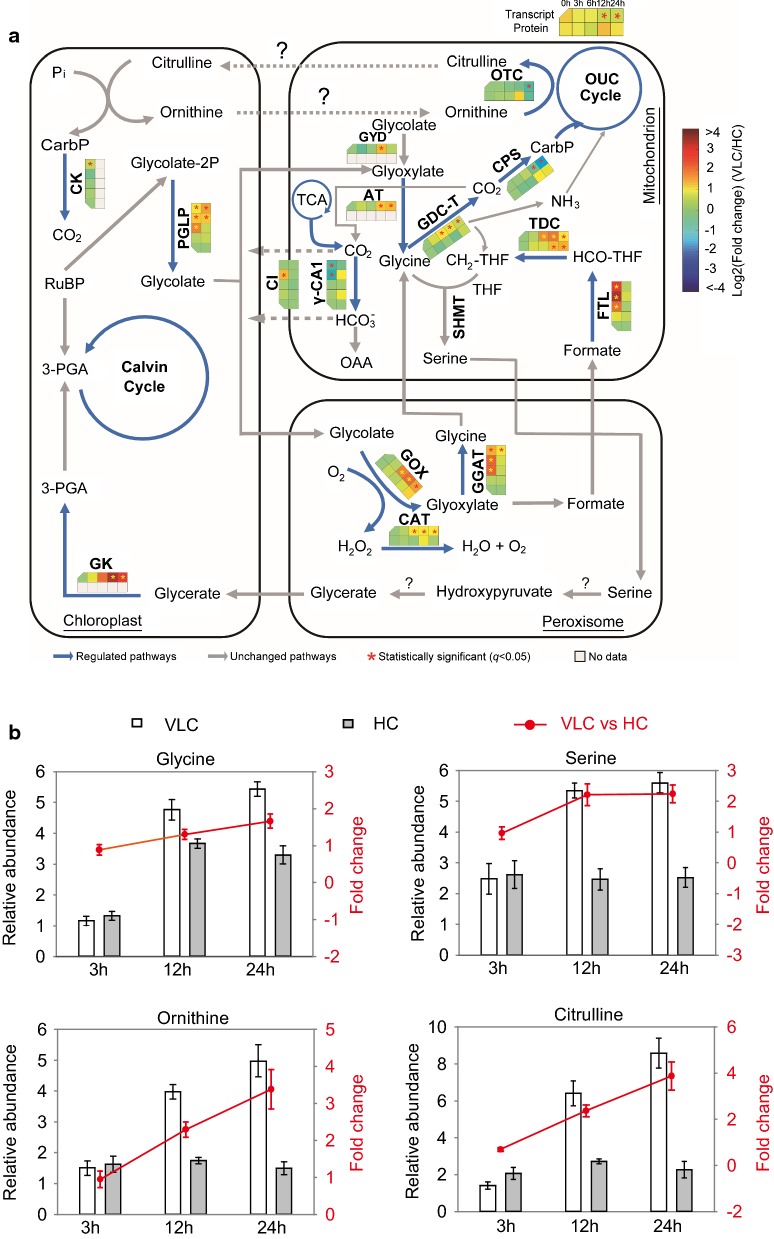
Basal CCM associated with the photorespiratory pathway, the one-carbon metabolism and the ornithine urea cycle in *N. oceanica* under VLC/HC. **a** Proposed “basal” CCM that is linked to the photorespiration pathway, one-carbon metabolism and the ornithine urea cycle. 3-PGA, 3-phosphoglyceric acid; CarbP, carbamoyl phosphate; CK, creatine kinase; CI, NADH–ubiquinone oxidoreductase complex I; γ-CA, γ-carbonic anhydrase; CPS, carbamoyl phosphate synthase; TDC, tetrahydrofolate dehydrogenase/cyclohydrolase; FTL, formyltetrahydrofolate ligase; GDC, glycine decarboxylase; GYD, glycolate dehydrogenase; OTC, ornithine transcarbamoylase; THF, tetrahydrofolate; OAA, oxaloacetate; TCA, tricarboxylic acid cycle; PGLP, phosphoglycolate phosphatase: GK, glycerate kinase; GOX, glycolate oxidase; GGAT, glutamate:glyoxylate aminotransferase; GDC-T, glycine decarboxylase complex T-protein; CAT, catalase; AT, alanine aminotransferase; SHMT, serine hydroxymethyltransferase. **b** Dynamics of the relative abundance of amino acids involved in photorespiration (glycine and serine) and the ornithine urea cycle (ornithine and citrulline)

In *N. oceanica*, a “basal” CCM is likely present, as suggested by upregulation of the key components under VLC (Fig. 7a): A NADH–ubiquinone oxidoreductase complex I (CI: g4915) that harbors a conserved CA domain (potentially interacting with mitochondrial carbonic anhydrase; [38, 40]) is upregulated 1.4-loget at 12 h under VLC/HC (Fig. 7a), yet downregulated (0.7–1.7-fold) under N−/N+ [21]; moreover, a γ-type CA transcript (g1084; with a mitochondrial signal peptide) is downregulated at 6 h under N−/N+ [21], yet exhibits a slight upregulation trend at 24 h under VLC/HC (Additional file 8: Dataset S1). Since excess CO_2_ would be produced in mitochondria as a result of two decarboxylation reactions (one from the TCA cycle and the other from glycine–serine conversion in photorespiration) under various stresses, this pathway can potentially recycle the portion of mitochondrial CO_2_ for the RuBisCO-mediated carbon fixation following CO_2_ diffusion into the chloroplast. Thus, this “basal” CCM in *N. oceanica* may be linked to the photorespiration pathway, which is highly compartmentalized as it involves chloroplasts, peroxisome, mitochondria and cytosol (Fig. 7a).

The reconstructed photorespiratory pathway of *N. oceanica*, similar to that of higher plants (i.e., the photorespiratory C2 cycle [41]; Fig. 7a), possesses several characteristic genes, like phosphoglycolate phosphatase (PGLP, g10166), glycerate kinase (GK, g1836), glycolate oxidase (GOX, g640), glutamate:glyoxylate aminotransferase (GGAT, g3637), glycine decarboxylase complex proteins (GDC, g176), alanine aminotransferase (AT, g6487) and serine hydroxymethyltransferase (SHMT; g1924 and g6217, with the latter being mitochondrion-targeted). In *Arabidopsis thaliana,* two genes encoding peroxisomal glutamate:glyoxylate aminotransferase (GGAT1, At1g23310 and GGAT2, At1g70580) participate in photorespiration; however, only one (g3637) is present in *N. oceanica* genome. Notably, two critical genes that are present in higher plant photorespiration, serine:glyoxylate aminotransferase (SGMT) and hydroxypyruvate reductase (HPR) are absent in *N. oceanica*, suggesting a yet-to-be defined photorespiratory pathway in this industrial microalga.

Under VLC/HC, almost all photorespiration genes in *N. oceanica* were strongly upregulated at 3 h, 6 h, 12 h and 24 h (Fig. 7a), including PGLP (g10166), GK (g1836), GOX (g640), AAT (g6487), GGAT (g3637) and SHMT (g6217, mitochondria). Notably, among the four units of glycine decarboxylase (GDC; H-, T-, L- and P-proteins; g260, g176, g1059 and g9801) only the T-protein transcript (g176; mitochondrion) was elevated by 1-loget at 12 h under VLC/HC. These imply that the photorespiratory carbon metabolism might play a decisive role in the re-assimilation of “lost carbon” via the oxygenation of RuBisCO under VLC. Moreover, the contents of glycine and serine increased by 1.3- and 1.7-loget under VLC/HC (Fig. 7b), consistent with the upregulation of photorespiratory CO_2_ cycle transcripts. At the protein level, GDC (g1059), GOX, GGAT and SHMT showed ca.1-2-loget upregulation at 24 h under VLC/HC (Fig. 7a).

The Wood–Ljungdahl pathway, usually found in bacteria and archaea, mediates anaerobic assimilation of CO and CO_2_; interestingly, homologous genes of this pathway are present in *N. oceanica*, e.g., formyltetrahydrofolate ligase (FTL, g5846), methylene tetrahydrofolate reductase (MTR, g3586 and g6732), tetrahydrofolate dehydrogenase/cyclohydrolase (TDC, g8540) and formaldehyde dehydrogenase (FDD, g5814); yet the formate dehydrogenase which catalyzes the first step reaction of this pathway is absent in the IMET1 genome. Although MTR (g3586 and g6732) was downregulated, FTL and TDC were highly upregulated (above 1.0-loget) under VLC/HC (Fig. 7a and Additional file 8: Dataset S1) and exhibited an expression pattern similar to the photorespiration genes (Additional file 9: Dataset S2), suggesting their participation in photorespiration and formation of a THF cycle to transfer –CH_3_ onto serine. Hence, in *N. oceanica* the incomplete Wood–Ljungdahl pathway may serve as a branch for photorespiratory carbon cycle.

In addition, a subset of genes in the ornithine urea cycle (OUC) was either upregulated [including ornithine aminotransferase (OAT; g2594)] or downregulated [e.g., carbamoyl phosphate synthase (CPS; g1666), ornithine transcarbamoylase (OTC, g1074), argininosuccinate synthase (ASS, g9579), argininosuccinate lyase (ASL; g281), arginase (ARG; g2649) and ornithine decarboxylase (ODC; g9591); Additional file 8: Dataset S1]. These genes (CPS and OTC) may also participate in the ornithine–citrulline shuttle between mitochondrion and chloroplast with creatine kinase (CK; g1246) [42]. The OUC [42] and the ornithine–citrulline shuttle are responsible for not only the re-assimilation of ammonia in mitochondrion, but CO_2_ transfer from mitochondrion to chloroplast. However, due to downregulation of the OUC and the ornithine–citrulline shuttle, CO_2_ from mitochondrion may not be timely transferred to the chloroplast. Therefore, the basal CCM might be the primary optional route for the transfer of CO_2_ (i.e., both products of the glycine decarboxylase reaction that need to be re-assimilated in the chloroplast) under VLC. Furthermore, photorespiration combined with the basal CCM could create a supplemental CO_2_ supply that enhances photosynthetic CO_2_ cycle.

Therefore, a “basal” CCM in IMET1 underpinned by the VLC-induced upregulation of photorespiration and the downregulation of ornithine–citrulline shuttle and the OUC appears to be present. Together the basal CCM and photorespiration are responsible for efficient recycling of mitochondrial CO_2_ for chloroplastic carbon fixation.

### Metabolic constraint of CCMs and photorespiration in *N. oceanica*: insights from comparative analysis with *C. reinhardtii* (Cr) and *P. tricornutum* (Pt)

Our results thus enabled the comparison of temporal transcriptomic and proteomic response to VLC conditions between the industrial microalga of *N. oceanica* and the laboratory model microalgae of *C. reinhardtii* (Cr) and *P.* *tricornutum* (Pt), which have been previously employed as laboratory models to study eukaryotic microalgal CCM [43]. Biophysical CCM is an important pathway of carbon flow under VLC. Not surprisingly, CAs and BCTs as representative components of the biophysical CCM are all present in Cr, Pt and *N. oceanica*. The Cr genome encodes at least 19 genes that encode CA isoforms, including three α, six β, four γ and four θ CAs [44]. In the Pt genome, there are at least 13 genes that encode CA isoforms, including five α, two β, two γ and four or θ CAs [45]. However, there are only five CA-encoding genes in *N. oceanica*. In addition, there are nine and two CAs regulated by rising or declining CO_2_ concentration in Cr and Pt, respectively, but only two CAs are regulated by CO_2_ availability in *N. oceanica* (Table 1). In Cr, several bicarbonate transporters have been characterized, including plasma membrane-localized HLA3 and LCI1, chloroplast envelop-localized LCIA, CCP1 and CCP2. In Pt, there are two kinds of bicarbonate transporters including SLC4 and SLC26 [45]. In comparison, there are merely two specific BCTs of the SLC4 family in *N. oceanica* (Table 1), although two potential homologs (g6826 and g532) of LCI1 are present (interestingly, g6826 was induced under VLC, implying another candidate BCT).

**Table 1:** Key genes from CCMs, C4-like metabolism and photorespiration in the laboratory model microalga *Chlamydomonas reinhardtii*, *Phaeodactylum tricornutum* and the model industrial oleaginous microalga *Nannochloropsis oceanica*

Gene or pathway	*Chlamydomonas reinhardtii*	*Phaeodactylum tricornutum*	*Nannochloropsis oceanica*
Carbonic anhydrase (CA)	CA1, CA2, CA3, CA4, CA5, CA6, CA7, CA8, CA9, CAG1, CAG2, CAG3	CA1, CA2, CA3, CA4, CA5, CA6, CA7, CA8, CA9, CA10, CA11, CA12, CA13	CA1, CA2, CA3, CA4, CA5 (i.e., CAH1)
Bicarbonate transporter	LCIB1, LCIB2, LCIB3	SLC4-1, SLC4-2, SLC4-3, SLC4-4, SLC4s, SLC26	BCT1, BCT2, homologs of LCIB1 (g6826 and g532)
C4-like genes	PEPC, PPDK1, PPDK2, ME, MDH	PEPC1, PEPC2, PEPCK, PPDK1, ME, MDH	PEPC, PEPCK, PPDK1, PPDK2, PPDK3, ME, MDH
Photorespiration	HPR, SMT, GS1, GS2	GS1, GS2, OUC cycle	GS1, GS2, OUC cycle

Genes induced by the low CO_2_ stress are highlighted by underlined font

CA, carbonic anhydrase; CAG, carbonic anhydrase in mitochondria; BCT, bicarbonate transporter; LCI, low-CO_2_-inducible protein; PEPC, phosphoenolpyruvate carboxylase; PEPCK, phosphoenolpyruvate carboxylase kinase; PPDK, pyruvate orthophosphate dikinase; MDH, malate dehydrogenase; ME, malic enzyme; SMT, serine:glyoxylate aminotransferase; HPR, hydroxypyruvate reductase; GS, glutamine synthetase

Although C4-like genes are present in Cr, Pt and *N. oceanica*, their expression patterns are very different in response to the stress of CO_2_ depletion (i.e., after the cells were transferred from high-carbon to low-carbon conditions). An increase in transcripts for phosphoenolpyruvate carboxylase (PEPC) and cytosolic NAD-dependent malate dehydrogenase was reported in Cr during CCM induction [46]. In Pt, its two PEPC transcripts were not affected by CO_2_ depletion [47]. In contrast, PEPC and MDH were not induced by carbon deficiency in *N. oceanica*. Surprisingly, PEPCK (a potential C4 photosynthesis decarboxylase) is absent in Cr and PEPCK transcript did not respond to CO_2_ depletion in Pt [47], but it was upregulated under VLC in *N. oceanica*. Additionally, both Cr and Pt harbor a chloroplastic PPDK and a cytosolic PPDK. However, there are three PPDK genes in *N. oceanica* and they are predicted as targeted to mitochondria, chloroplast and cytosol, respectively. While neither of the two PPDK genes in Cr and Pt was regulated by the CO_2_ level [17, 48], the PPDK genes in *N. oceanica* were all greatly upregulated under VLC. These profound differences in both genetic reservoir and gene expression imply a CCM system in *N. oceanica* that is relatively simple in structure (e.g., the fewer number of CAs) yet finely regulated in function, as compared to those of Cr and Pt (Table 1).

Photorespiration is another important pathway for controlling carbon flow. Because the knowledge of photorespiration in Pt is limited, we only compared the difference between Cr and *N. oceanica*. Though the photorespiratory carbon cycle is enhanced in both Cr and *N. oceanica* (as revealed by the transcriptomic and metabolomic dynamics after the transfer of microalgal cells to low CO_2_), distinctions are apparent between the two microalgae: *firstly*, HPR and SMT which are present in photorespiration of Cr are absent in that of *N. oceanica*. Interestingly, the brown alga *Ectocarpus siliculosus* also lacks these two genes [49], underscoring the similarity of photorespiration pathway between *N. oceanica* and *E. siliculosus*; *secondly*, as photorespiration is activated and the carbon flux through the Gly decarboxylate complex increases, in the mitochondria release of NH_3_ would occur, which has to be recaptured since excessive accumulation of ammonia would result in cell damage [50]. Interestingly, the approaches for such ammonia dislodgment are different. In Cr, ammonia is re-fixed by glutamine synthetase (GS), as both the cytosolic GS1 and the chloroplastic GS2 gene expression increase [17, 48]. However, in *N. oceanica*, GS was not upregulated under VLC (Table 1); instead, the ornithine shuttle associated with OUC was upregulated to recapture the ammonia (protein abundance; Fig. 7a; Additional file 9: Dataset S2; notably, the OUC is absent in Cr). Taken together, there are also profound distinctions between *N. oceanica* and *C*r in the CCM-associated photorespiration metabolism.

## Conclusion

For the model industrial oleaginous microalga *N. oceanica*, dynamics of transcriptome, proteome and metabolome that underlie cellular adaption from high CO_2_ level to very low CO_2_ revealed a massive reprogramming of cellular metabolism. The activity of a biophysical CCM is evidenced based on induction of transcripts encoding a bicarbonate transporter and two carbonic anhydrases under VLC. Moreover, the presence of a potential biochemical CCM is supported by the upregulation of a number of key C4-like pathway enzymes in both protein abundance and enzymatic activity under VLC, consistent with a mitochondria-implicated C4-based CCM. Furthermore, a basal CCM underpinned by VLC-induced upregulation of photorespiration and downregulation of ornithine–citrulline shuttle and the OUCs is likely present, which may be responsible for efficient recycling of mitochondrial CO_2_ for chloroplastic carbon fixation. Therefore, *N. oceanica* appears to mobilize a comprehensive set of CCMs in response to low carbon stress. The specific genes induced under the very low level of CO_2_ are quite distinct from those of *C. reinhardtii* and *P.* *tricornutum*, suggesting tightly regulated yet rather unique CCMs in this organism. These VLC-induced genes, such as CAs, PEPCK, ME and PPDK, are promising targets for functional validation and for exploitation for strain development [28, 51]. Therefore, the findings in this study can serve as the first step for rational engineering of the CCMs for enhanced carbon fixation and biomass productivity in industrial oleaginous microalgae.

## Materials and methods

### Culture conditions of *N. oceanica*

*Nannochloropsis oceanica* IMET1 was inoculated into the modified f/2 liquid medium, which was prepared with 35 g L^−1^ sea salt (Real Ocean, USA), 1 g L^−1^ NaNO_3_, 67 mg L^−1^ NaH_2_PO_4_*H_2_O, 3.65 mg L^−1^ FeCl_3_*6H_2_O, 4.37 mg L^−1^ Na_2_EDTA*2H_2_O, trace metal mix (0.0196 mg L^−1^ CuSO_4_*5H_2_O, 0.0126 mg L^−1^ NaMoO_4_*2H_2_O, 0.044 mg L^−1^ ZnSO_4_*7H_2_O, 0.01 mg L^−1^ CoCl_2_ and 0.36 mg L^−1^ MnCl_2_*4H_2_O) and vitamin mix (2.5 µg L^−1^ VB_12_, 2.5 µg L^−1^ biotin and 0.5 µg L^−1^ thiamine HCl) [52]. The cells were first cultured in f/2 medium at 25 °C with 80 ± 5 μmol m^−2^ s^−1^ continuous irradiation in a 1-L column reactor (inner diameter 5 cm). The seed cultures were bubbled with 5% CO_2_. At the logarithmic phase (OD_750_ = 3.0), cells were harvested by centrifugation and then washed with fresh medium, before being used for the following experiments.

In total, six identical column reactors were employed for the wild-type *N. oceanica* culture. Each reactor contains 800 mL of fresh modified f/2 liquid medium, which was supplemented with 10 mM Tris–HCl buffer (pH = 8.2) in order to accurately control the pH during the culture. Equal numbers of the seed cells from six independent reactors were re-inoculated into each of the six new column reactors with fresh medium to an OD_750_ of 1.5, respectively. The light intensity was maintained at 80 ± 5 μmol m^−2^ s^−1^. The six algal cultures were first aerated with air enriched with 5% CO_2_ (“high-CO_2_” conditions, or HC) for 1 h. After the preadaption phase, three of the algal cultures proceeded under HC as the control condition, whereas the other three were switched to aeration with 0.01% CO_2_ (“very low-CO_2_” conditions, or VLC; the customized CO_2_ gas was provided by Dehai Gas Company, China) for CCM induction (Additional file 1: Figure S1; [17, 48]). After switching to the designated culture condition (e.g., VLC), cell aliquots were taken at 0, 3, 6, 12 and 24 h from each column by syringe for physiological measurement (including OD, inorganic carbon concentration, chlorophyll content, photosynthetic rate, etc.), transcriptomic profiling, proteomic profiling and metabolite analysis. Three biological replicates of algal cultures, corresponding to the collectively six column reactors, were established under each of the above VLC and HC conditions, respectively.

### Tracking the photosynthetic activity of *N. oceanica*

Chlorophyll fluorescence parameters sensitively reflect the instantaneous photosynthetic state of microalgae and their acclimation to current environmental conditions [53]. Fv/Fm (the variable/maximum fluorescence ratio), the maximum photochemical quantum yield of PSII reaction centers, represents the minimum fluorescence yield when PSII reaction centers are fully open and reflects the photosynthetic light energy conversion efficiency. On the other hand, Fv′/Fm′ represents the active PSII activity; therefore, Fv/Fm and Fv′/Fm′ are both measured here to depict photosynthetic performance and acclimation status [54]. Fm is the maximum fluorescence yield when PSII reaction centers are completely closed; thus, it reflects the PSII electron transport capacity. Fv is the variable fluorescence (Fv = Fm − Fo), reflecting reduction in the PSII primary electron acceptor QA, thus indicating the photochemical activity of PSII reaction centers. Fo is the minimum fluorescence yield. (Damage to or irreversible loss of activity of PSII reaction centers will cause a decrease in the Fo value.) Fv/Fm and Fv′/Fm′ were calculated according to these two formulas Fv/Fm = (Fm − Fo)/Fm and Fv′/Fm′ = Fm′ − Ft/Fm′ [53]. To measure these parameters, *N. oceanica* cultures were kept in the dark for 20 min and then exposed to a saturating light pulse (1000 mol m^−2^ s^−1^) for l s, while the chlorophyll fluorescence intensities were measured with a pulse amplitude-modulated (PAM) kinetics using IMAGING-PAM M-Series (Walz, Germany) following the manufacturer’s recommendations.

### Measurement of total inorganic carbon content in the medium

Total dissolved inorganic carbon content (TIC) in the medium was measured using a high-temperature TOC/TNb analyzer (LiquiTOC II, Elementar, Germany) coupled with automatic sampling instrument [55]. To prepare for the measurement, 3 mL algal medium was diluted 10 times with the distilled Milli-Q water and transferred into a 30 mL brown glass reagent bottle. Another 3 mL medium filtered by pre-combusted GF/F filter (0.7 µm pore size, 25 mm) was diluted 10 times with distilled water and then transferred to a 30 mL brown glass reagent bottle. Both samples were acidified with 100 µL nitric acid and then stored at 20 °C for the measurement.

### Measurement of lipid, carbohydrate and protein

To quantify the amount of carbohydrate, protein and lipid, *N. oceanica* cells were harvested after 24 h cultivation by centrifugation (at 4000 rpm for 5 min) under VLC and HC. For dry algal powder, cells were lyophilized for 2 days. Extraction and assaying of lipid, carbohydrate and protein in the microalgal biomass were performed based on our published protocols [56].

### Metabolite analysis by GC–mass spectrometry

A 10 mg (DW) sample of the microalgal biomass, which had been frozen in liquid nitrogen and stored at − 80 °C, was extracted for metabolite analysis according to Lisec et al. with slight modifications [57]. Lyophilized algal culture was carefully weighted to about 5.00 mg and transferred to a microcentrifuge tube. 500 μL of 100% (v/v) methanol supplemented with 2 μg of ribitol for sample normalization was added to the algae powder. Metabolite extraction was performed by 15 min of shaking in a thermomixer (1200 rpm) at 70 °C. Cell debris was centrifuged at 16,000×g for 5 min, and 100 μL of the supernatant solution was dried in a vacuum evaporator for 3 h. Dried samples were derivatized by the addition of 20 μL of a 20 mg/mL solution of methoxylamine hydrochloride (Sigma-Aldrich, USA) in pyridine (30 °C for 90 min). 30 μL of *N*-methyl-*N*-(trimethylsilyl) trifluoroacetamide (MSTFA) was then added and shaken for a further 30 min at 37 °C. Totally 10 μL of an alkane standard mix containing 50 ng each of C12, C15, C19, C22, C28, C32 and C36 in chloroform was added for retention index determination with high accuracy [58, 59]. Samples were randomized, and 1 μL of derivatized sample was injected splitless into an Agilent 6890 GC fitted with an Agilent 5975 MSD. Helium was used as the carrier gas at a constant flow of 1 ml/min. Inlet temperature was set at 300 °C. Oven temperature was initially set at 70 °C for 1 min, ramped at 1 °C/min until 76 °C and then ramped at 6 °C/min until 325 °C, with a final hold of 10 min. A Varian Factor 4 capillary column (VF-5 ms, 30 m × 0.25 mm, 0.25 μm plus 10 m EZ-Guard) was used. The MSD transfer line heater was kept at 300 °C. MS quadrupole temperature was kept at 150 °C and source temperature at 230 °C. Mass detection range was set from 40 to 600 atomic mass units. Spectral data files were processed with AMDIS (version 2.65) for metabolite identification. Metabolites were identified by retention index and spectral comparison with pre-run standards or by searching the NIST library. All identified metabolites were entered into MSD ChemStation (version E.02.00.493), and a quantitation database was created using specific target ions and qualifier ions unique to each metabolite. All spectra were manually reviewed. Normalization was performed to the internal standard ribitol and to the tissue weight. Student’s t test was used to compare the two datasets (VLC and HC, *n* = 6) at the same time point. If the test gave a *P* value ≤ 0.05, the difference between VLC and HC was interpreted as being significant.

### Transcriptome sampling, sequencing and analysis

For transcriptomic analyses, the cells were harvested by centrifugation for 5 min at 2500*g* and then were immediately quenched with liquid N_2_ and stored in − 80 °C freezer. Total algal RNA was extracted using Trizol reagents (Invitrogen, USA). The concentration and purity of the RNA were determined spectrophotometrically (NanoDrop-1000, Thermo Scientific, USA).

For mRNA-Seq, the poly (A)-containing mRNA molecules were purified using Sera-Mag Magnetic Oligo (dT) Beads (Thermo Scientific, USA) and were fragmented into 200- to 300-bp fragments by incubation in RNA Fragmentation Reagent (Ambion, USA) according to the manufacturer’s instructions. The fragmented mRNA was then purified from the fragmentation buffer using Agencourt^®^ RNA Clean beads (Beckman Coulter, USA). The purified, fragmented mRNA was converted into double-stranded cDNA using the SuperScript Double-Stranded cDNA Synthesis Kit (Invitrogen, USA) by priming with random hexamers. Strand nonspecific transcriptome libraries were prepared using the NEBNext^®^ mRNA Library Prep Reagent Set (New England Biolabs, USA) and sequenced for 2 × 90 bp runs (paired-end, PE) using Illumina HiSeq 2000.

To ensure quality, the raw data (2 × 90 bp PE reads) were modified as follows: First, adapter pollutions in reads were deleted, and then, because the sequence qualities of Illumina reads degrade quickly toward the 3′ end, all reads were trimmed from the 3′ end until the 3′-end–most position with Phred equivalent score was 20 or greater. The raw data were deposited in NCBI GEO with the reference series number GSE55861. These filtered Illumina reads were aligned to our previously published *N. oceanica* IMET1 genome [20] with TopHat (version 2.0.4, allowing no more than two segment mismatches) [60]. Reads mapped to more than one location were excluded. Thirdly, the short read mapping results from TopHat were used for the differential gene expression analysis with Cufflinks (version 2.0.4), as was described [61].

For each of the mRNA-Seq datasets under each experimental condition, gene expression was measured as the numbers of aligned reads to annotated genes by Cufflinks (version 2.0.4) and normalized to FPKM values (fragments per kilobase of exon model per million mapped fragments). Genes were considered to be significantly differentially expressed if either of the conditions was met: (i) Their expression values showed at least twofold change with a false discovery rate (FDR)-corrected *p* value ≤ 0.05 (provided by Cuffdiff from the Cufflinks package) between control and stressed conditions, and moreover, their FPKM values at either condition were ≥ 10. (ii) Their expression values showed 1.5- to less than twofold change with a FDR-adjusted *p*-value ≤ 0.05 between control and stressed conditions for at least two time points, and moreover, their FPKM values at either of the conditions were ≥ 10.

The 2933 differentially expressed genes were grouped into 16 clusters based on their temporal expression patterns by the *k*-means clustering using the Multiple Experiment Viewer 4.8 (MeV4.8; http://www.tm4.org/mev/) with the Euclidean distance [62]. The optimal number of clusters was identified and investigated by performing a figure of merit (FOM) analysis within MeV4.8 [63]. FOM analysis showed that the value was stabilized after a partitioning into 12–18 clusters using *k*-means algorithm. Therefore, the transcripts were split into 16 clusters, each of which exhibits a particular pattern of temporal dynamics.

### Validation of transcript abundance using Real-time qPCR

To further test the validity of the mRNA-Seq results, RNA extracted from the same cultures for mRNA-Seq was subjected to the PrimeScript^®^ RT reagent Kit with gDNA Eraser (Takara, Japan) for cDNA synthesis. Also, qRT-PCR was performed by standard methods (Roche, Switzerland) as previously described [64]. Ct values were determined for triplicate independent technical experiments performed on triplicate biological cultures (*n* = 3). Relative fold differences were calculated based on the *ΔCt* method using the *actin* amplification product as an internal standard. Primer pairs used for qRT-PCR analyses are listed in Additional file 5: Table S3. Sizes of amplification products were 100 to 300 bp. The correlation coefficient between the qPCR results and the mRNA-Seq results for the 12 genes tested was 0.94 (*R*^2^; Additional file 6: Figure S3).

### Proteome sampling, sequencing and analysis

*Nannochloropsis oceanica* cells were collected by centrifugation at 2500 g at 4 °C. Total proteins were extracted with plant protein extraction kit (CWBIO, Beijing) and quantified by the approach of BCA protein assay (CWBIO, Beijing). Protein samples were loaded onto 12.5% (v/v) polyacrylamide gels containing 0.4% (w/v) SDS (50–100 µg sample per lane). The gels were run at room temperature, 300 V and 30 mA until all proteins migrated about 1 cm into the separation gel Proteins were visualized with a coomassie brilliant blue (CBB-G250) stain as previously described [65]. Protein bands were excised from the gels, cut into small cubes (ca. 1 × 1 mm^3^) and destained [66]. Gel pieces were dried in a SpeedVac and immersed completely in digestion solution (~ 200 µL). The digestion solution consisted of sequencing grade modified trypsin (Promega, USA), which was diluted in 40 mM ammonium bicarbonate (pH 8.6) to a concentration of 12.5 ng µL^−1^.

The protein digestion was performed overnight at 37 °C with tempered shaker (HLC MHR20, 550 rpm). After digestion, the samples were centrifuged and supernatants were transferred to LC–MS grade glass vials (12 × 32 mm^2^ glass screw-necked vial, Waters, USA). The extracted peptides were dried using a SpeedVac and stored at room temperature. Prior to MS analysis, peptides were re-suspended in 20 µL of buffer A (0.1% formic acid in water, ULC/MS; Biosolve, the Netherlands) by sonication for 10 min and used for MS analysis. Each measurement was taken with 8 μL of sample.

An UPLC HSS T3 column (1.8 mm, 75 mm, 150 mm, Waters, USA) and an UPLC Symmetry C18 trapping column (5 mm, 180 mm, 20 mm, Waters, USA) for LC as well as a PicoTip Emitter (SilicaTip, 10 mm i.d., New Objective, USA) were used in combination with the nanoACQUITY gradient UPLC pump system (Waters, USA) coupled to a LTQ Orbitrap Elite mass spectrometer (Thermo Scientific, USA). For elution of the peptides, a gradient with increasing concentration of buffer B (0.1% formic acid in acetonitrile, ULC/MS, Biosolve, the Netherlands) was used in 105 min at a flow rate of 400 nL/min and a spray voltage of 1.6 kV: 0–5 min: 2% buffer B; 5–10 min: 2–5% buffer B; 10–71 min: 5–30% buffer B; 72–77 min: 85% buffer B; 77–105 min: 2% buffer B. The analytical column oven was set to 55 °C, and the heated desolvation capillary was set to 275 °C. The LTQ Orbitrap Elite was operated via instrument method files of Xcalibur (Rev. 2.1.0) in positive ion mode. The linear ion trap and Orbitrap were operated in parallel, i.e., during a full MS scan on the Orbitrap in the range of 150–2000 *m/z* at a resolution of 60,000 MS/MS, spectra of the 20 most intense precursors were detected in the ion trap using the rapid scan mode. The relative collision energy for collision-induced dissociation (CID) was set to 35%. Dynamic exclusion was enabled with a repeat count of 1 and a 45 s exclusion duration window. Singly charged ions of unknown charge state were rejected from MS/MS.

Protein identification for VLC and HC data was performed with Proteome Discoverer using Sequest HT. Protein identification for wild-type data was performed by Andromeda search engine [67] embedded in MaxQuant [68, 69], which searched against the complete proteome database of *N. oceanica* IMET1 [20]. The mass tolerance for precursor ions was set to 10 ppm; the mass tolerance for fragment ions was set to 0.4 Da. Only tryptic peptides with up to two missed cleavages were accepted. The oxidation of methionine, acetylation on N-terminal and propionamide on cysteine was admitted as a variable peptide modification. The false discovery rate (FDR, *q* value) of protein identification was set to 1% and was determined with the percolator validation in Proteome Discoverer (for VLC and HC).

For the VLC versus HC comparison, un-normalized PSM values were imported into Perseus software. After a log2 transformation, the samples were normalized on the median value of each sample for comparability. Then a two-way ANOVA was employed as mentioned for wild-type data analysis. For each time point, a two-sample *t* test was conducted with a S0 value of 0.1 and an FDR of 0.05; a *q* value was also reported during this analysis. All the other analyses were carried out in the MATLAB^®^ environment for statistical computing and graphics.

It has been well recognized that correlation between proteomic data and transcriptomic data is not necessarily high, i.e., 50% [70, 71]; moreover, protein expression is usually delayed in plants as compared to transcriptomic data [72]. Therefore, here in most cases, the more comprehensive transcriptome data were exploited for further analyses. However, important findings were supported by both transcriptomics and proteomics, e.g., the strongly increased expression of CA5 in biophysical CCM and PPDK2 in biochemical CCM.

### Predicting the subcellular localization of proteins

To determine possible compartmentalization of CCMs, central carbon metabolism, photorespiration metabolism and OUC in IMET1, a series of software was used to predict their signals of subcellular localization. Firstly, SignalP was used to predict secretory signal peptide which targets its passenger protein for translocation across the endoplasmic reticulum membrane in eukaryotes [73]. Secondly, ChloroP prediction was performed, which presents a neural network-based method for identifying chloroplast transit peptides and their cleavage sites [74]. Thirdly, the program MitoProt was employed to evaluate mitochondrial targeting signals, which is suitable for studying mitochondria-related proteins [75]. Lastly, HECTAR was used to predict their subcellular localization signals [76]. HECTAR is able to predict the subcellular localization of heterokont proteins with high accuracy and to assign proteins to five different categories of subcellular targeting including signal peptides, type II signal anchors, chloroplast transit peptides, mitochondrion transit peptides and proteins which do not possess any N-terminal target peptide. Results from the four programs were pooled and those with majority consensus were chosen as the predicted localization for a particular protein.

### Measurement of the enzymatic activity of key CCM genes

Crude enzyme extracts were prepared from the algal powder in three times the volume of ice-cold extraction buffer. Enzyme activities were determined spectrophotometrically using a UV-1800 spectrophotometer by measuring at 340 nm in total volumes of 0.4 mL and in triplicate. The change in absorbance was recorded for 5 min. Specifically, for every 100 mg fresh algal powder, 300 μL of chilled extraction buffer (40 mM Tris-HC1, 0.25 mM EDTA, 10 mM MgCl_2_, 5 mM glutathione, at pH 7.6) was added. The mixture was stirred for 2 min to homogeneity and centrifuged at 13,000*g* for 10 min. The supernatants were incubated on ice for further enzyme assays. The enzymatic activities of PEPC, PEPCK, NAD-ME, NADP-ME, MDH and RuBisCO were measured using quantification kits (Keming Biotech, China). The total protein content was quantified using BCA protein assay kit (Thermo Scientific, USA). The activity of different enzymes was calculated based on the content of total protein, with the activity unit defined as μmol NAD(P)H oxidation or NAD(P)+ reduced per minute in total protein (min^−1^ mg^−1^) [77].

### Immunoblot assay for the quantification of PEPCK protein

Total cellular proteins were extracted from 10 to 20 mg DW IMET1 under LVC and HC conditions using the Pierce™ P-PER plant protein extraction kit (Thermo Scientific, USA). Western blot analyses were performed with total protein from cell extracts after resolution by sodium dodecyl sulfate–polyacrylamide gel electrophoresis (SDS-PAGE) using a 12% (w/v) acrylamide resolving gel (Bio-Rad, USA). Sample loading was based on equal total protein (15 µg). The separated proteins were transferred to a polyvinylidene difluoride (PVDF) membrane, and nonspecific antibody binding was blocked with 5% (w/v) nonfat dried milk in Tween 20–phosphate-buffered saline (TBS; pH 7.4) for 1 h at room temperature. The membranes were then incubated overnight at 4 °C with polyclonal anti-PEPCK (AS10700) antibodies [78] from Agrisera diluted 1:10,000 in phosphate-buffered saline (PBS) containing 1% (w/v) nonfat milk. After washing, the membranes were incubated with goat anti-rabbit IgG–horseradish peroxidase (HRP) secondary antibody. Protein bands were visualized using a solution containing 3,3′-diaminobenzidine tetrahydrochloride as the peroxidase substrate, and the membranes were scanned.

## Additional files

**Additional file 1: Figure S1.** A schematic diagram of the experimental design. *Nannochloropsis oceanica* cells were firstly grown to logarithmic phase under air enriched with 5% CO_2_. After adaption to new environment under 5% CO_2_ for an hour, the cells were then cultured under either 100 pm or 50,000 ppm CO_2_ concentration. Samples were collected at 0, 3, 6, 12 and 24 h from each condition (three biological replicate columns for each) by syringe for physiological characterization and multi-omics (transcriptome, proteome and metabolome) profiling.

**Additional file 2: Figure S2.** Growth curves and photosynthetic efficiency of *N. oceanica* IMET1 cells under VLC and HC conditions. (**A**) Growth curve of *Nannochloropsis* under two CO_2_ concentrations. The y-axis presents the average optical densities of triplicate algal cultures at 750 nm at each time point. Data are averages of at least three independent experiments (error bars represent standard deviations). (**B**) Maximum quantum efficiency (Fv/Fm) and active activity (Fv’/Fm’) of Photosystem II under VLC and HC conditions. The sharp decrease in Fv/Fm observed at 3h might be caused by short-term acclimation of the microalga to low carbon. (**C**) Change in concentration of total dissolved inorganic carbon (DIC) in the medium under VLC and HC conditions. The rapid increase in DIC for cells cultivated under HC was due to the start of aeration with 5% CO_2._

**Additional file 3: Table S1.** General information on the time series mRNA-Seq and proteome datasets under VLC and HC.

**Additional file 4: Table S2.** Spearman correlation of the global transcriptome profiles of the wild-type *N. oceanica* under VLC/HC (Excel file).

**Additional file 5: Table S3.** Real-time PCR primer sequences for the 12 genes used in real-time PCR experiments to validate the mRNA-Seq results.

**Additional file 6: Figure S3.** Validation of mRNA-Seq-based transcript quantification using real-time quantitative PCR (qPCR). Twelve genes involved in CCM and photorespiration metabolism are selected for qPCR validation. The genes and qPCR primer sequences are listed in Additional file 5: Table S3. The transcript levels at each time point after the onset of carbon limitation are included, and the correlation coefficient between the average qPCR-based transcript abundance and the mRNA-Seq-based transcript abundance is 0.9497 (R^2^).

**Additional file 7: Figure S4.** Transcript dynamics of the genes related to CCM and photorespiration metabolism in *N. oceanica* IMET1 as measured by mRNA-Seq and real-time quantitative PCR. Each data point represents the average of three biological replicates. Each sample was analyzed in technical triplicates.

**Additional file 8: Dataset S1.** Transcriptome dynamics of the wild-type *N. oceanica* under the VLC and HC conditions and manual functional curation of the transcripts (Excel file). The 2933 differentially expressed genes are grouped into 16 clusters based on their temporal patterns of transcript abundance. Variation of transcript abundance as indicated by the log_2_(VLC/HC) along time course of genes in each cluster is listed. Each of the 16 gene clusters in Fig. 1b is provided as a separate excel sheet. The mRNA-Seq data for the genes involved in the CCMs and the photorespiration metabolisms are listed as an additional sheet (the last sheet).

**Additional file 9: Dataset S2.** Proteome dynamics of the wild-type *N. oceanica* under the VLC and HC conditions. Among the 1965 proteins identified, the 255 differentially expressed proteins are grouped into six clusters based on temporal pattern of protein abundance. For each cluster in **Figure 2B**, variation of protein abundance as indicated by log_2_(VLC/HC) along the time course is provided as a separate Excel sheet.

**Additional file 10: Dataset S3.** Dynamics of cellular metabolites of the wild-type *N. oceanica* under the VLC and HC conditions (Excel file). Average relative cellular contents at both conditions along time course are listed for each species. The variations are indicated by fold change.

**Additional file 11: Figure S5.** Contents of carbohydrates, proteins and lipids under VLC and HC. The biochemical compositions in the microalga biomass were measured under VLC and HC. Data are shown as means ± SD of three replicates.

**Additional file 12: Table S4.** Predicted subcellular localization of *N. oceanica* IMET1 genes related to biophysical CCM, the C4-like cycle, the photorespiratory metabolism, the THF cycle and the ornithine urea cycle (Excel file). The analysis is based on predictions from various computational programs (“Materials and methods”). Abbreviations: Y, Yes; -, not detected; C, chloroplast; M, mitochondria; O, other; SP, secretory pathway; P, peroxisome.

**Additional file 13: Figure S6.** Proposed response of central carbon metabolism during CCM induction in *N. oceanica* IMET1. 1,3PG, 1,3-biphosphoglycerate; 2OG, α-ketoglutarate; 2PG, 2-phosphoglycerate; 3PG, 3-phosphoglycerate; AAT, ADP/ATP transporter; ACS, acetyl-CoA synthetase; ACT, aconitate; AHD, aconitate hydratase; ALDO, fructose-1,6-bisphosphate aldolase; CS, ATP citrate synthase; DHAP, dihydroxyacetone phosphate; ENL, enolase; ENR, enoyl-ACP reductase; F1,6P, fructose 1,6-biphosphate; FBP, fructose bisphosphatase; F6P, fructose-6-phosphate; FHD, fumarate hydratase; FUM, fumarate; G6P, glucose-6-phosphate; GK, glucose kinase; Glu, glucose; GPDH, glyceraldehyde-3-phosphate dehydrogenase; GPI, glucose phosphate isomerase; GS, 1,3-β-glucan synthase; HAD, hydroxyacyl-ACP dehydrogenase; ICDH, isocitrate dehydrogenase; ICIT, isocitrate; MHD, malate dehydrogenase; NTT, nucleotide transporter; OGDH, 2-oxoglutarate dehydrogenase; PGAM, phosphoglycerate mutase; PGK, phosphoglycerate kinase; PFK, phosphofructose kinase; PPDK, phosphate dikinase; PPT, Pi/PEP translocator; PYR, pyruvate; SCS, succinyl-CoA synthetase; SDH, succinate dehydrogenase; SUC, succinyl-CoA; TPI, triosephosphate isomerase; SFC, succinate/fumarate carrier; FBPA: fructose-1,6-bisphosphate aldolase; TL: transketolase; SBP: sedoheptulose bisphosphatase; RPI: ribose-5-phosphate isomerase; RPE: ribulose-phosphate 3-epimerase. The metabolites up- or downregulated based on the metabolomics analysis are indicated by red arrows.

## Data Availability

All data generated or analyzed during this study are included in its additional files.

## Abbreviations

CA: carbonic anhydrase
BCT: bicarbonate transporter
CER: chloroplast endoplasmic reticulum
PPC: periplastidal compartment
PEPC: phosphoenolpyruvate carboxylase
PEPCK: phosphoenolpyruvate carboxylase kinase
PPDK: pyruvate orthophosphate dikinase
MDH: malate dehydrogenase
ME: malic enzyme
PK: pyruvate kinase
PYC: pyruvate carboxylase
PPT: Pi/PEP translocator
SFC: succinate/fumarate carrier
OAA: oxaloacetate
CIT: citrate
FUM: fumarate
SUC: succinate
α-KG: α-ketoglutarate
PEP: phosphoenolpyruvate
PYR: pyruvate
MAL: malate
3-PGA: 3-phosphoglyceric acid
CarbP: carbamoyl phosphate
CK: creatine kinase
CI: NADH–ubiquinone oxidoreductase complex I
γ-CA: γ-carbonic anhydrase
CPS: carbamoyl phosphate synthase
TDC: tetrahydrofolate dehydrogenase/cyclohydrolase
FTL: formyltetrahydrofolate ligase
GDC: glycine decarboxylase
GYD: glycolate dehydrogenase
OTC: ornithine transcarbamoylase
THF: tetrahydrofolate
OAA: oxaloacetate
TCA: tricarboxylic acid
PGLP: phosphoglycolate phosphatase
GK: glycerate kinase
GOX: glycolate oxidase
GGAT: glutamate:glyoxylate aminotransferase
GDC: glycine decarboxylase complex
CAT: catalase
AT: alanine aminotransferase
SHMT: serine hydroxymethyltransferase

## References

[1] Beaugrand G, Edwards M, Legendre L (2010). Marine biodiversity, ecosystem functioning, and carbon cycles. Proc Natl Acad Sci USA.

[2] Thomas MK, Kremer CT, Klausmeier CA, Litchman E (2012). A global pattern of thermal adaptation in marine phytoplankton. Science.

[3] Tatters AO, Roleda MY, Schnetzer A, Fu F, Hurd CL, Boyd PW, Caron DA, Lie AA, Hoffmann LJ, Hutchins DA (2013). Short- and long-term conditioning of a temperate marine diatom community to acidification and warming. Philos Trans R Soc Lond B Biol Sci.

[4] Kumar K, Dasgupta CN, Nayak B, Lindblad P, Das D (2011). Development of suitable photobioreactors for CO_2_ sequestration addressing global warming using green algae and cyanobacteria. Bioresour Technol.

[5] Ugwu CU, Aoyagi H, Uchiyama H (2008). Photobioreactors for mass cultivation of algae. Bioresour Technol.

[6] Sydney EB, Sturm W, de Carvalho JC, Thomaz-Soccol V, Larroche C, Pandey A, Soccol CR (2010). Potential carbon dioxide fixation by industrially important microalgae. Bioresour Technol.

[7] NAABB final report. United States Department of Energy, Office of energy efficiency and renewable energy, National Alliance for Advanced Biofuels and Bioproducts (NAABB) Final Report. http://wwwenergygov/eere/bioenergy/downloads/national-alliance-advanced-biofuels-and-bioproducts-synopsis-naabb-final. 2014.

[8] Long BM, Rae BD, Rolland V, Forster B, Price GD (2016). Cyanobacterial CO_2_-concentrating mechanism components: function and prospects for plant metabolic engineering. Curr Opin Plant Biol.

[9] Reinfelder JR (2011). Carbon concentrating mechanisms in eukaryotic marine phytoplankton. Ann Rev Mar Sci.

[10] Heyduk K, Moreno-Villena JJ, Gilman IS, Christin PA, Edwards EJ (2019). The genetics of convergent evolution: insights from plant photosynthesis. Nat Rev Genet.

[11] Hopkinson BM, Dupont CL, Matsuda Y (2016). The physiology and genetics of CO_2_ concentrating mechanisms in model diatoms. Curr Opin Plant Biol.

[12] Mackinder LCM (2018). The *Chlamydomonas* CO_2_-concentrating mechanism and its potential for engineering photosynthesis in plants. New Phytol.

[13] Reinfelder JR, Kraepiel AM, Morel FM (2000). Unicellular C4 photosynthesis in a marine diatom. Nature.

[14] Klodmann J, Sunderhaus S, Nimtz M, Jansch L, Braun HP (2010). Internal architecture of mitochondrial complex I from *Arabidopsis thaliana*. Plant Cell.

[15] Meyer M, Griffiths H (2013). Origins and diversity of eukaryotic CO_2_-concentrating mechanisms: lessons for the future. J Exp Bot.

[16] Clement R, Jensen E, Prioretti L, Maberly SC, Gontero B (2017). Diversity of CO_2_-concentrating mechanisms and responses to CO_2_ concentration in marine and freshwater diatoms. J Exp Bot.

[17] Brueggeman AJ, Gangadharaiah DS, Cserhati MF, Casero D, Weeks DP, Ladunga I (2012). Activation of the carbon concentrating mechanism by CO_2_ deprivation coincides with massive transcriptional restructuring in *Chlamydomonas reinhardtii*. Plant Cell.

[18] Ewe D, Tachibana M, Kikutani S, Gruber A, Rio Bartulos C, Konert G, Kaplan A, Matsuda Y, Kroth PG (2018). The intracellular distribution of inorganic carbon fixing enzymes does not support the presence of a C4 pathway in the diatom *Phaeodactylum tricornutum*. Photosynth Res.

[19] Reinfelder JR, Milligan AJ, Morel FM (2004). The role of the C4 pathway in carbon accumulation and fixation in a marine diatom. Plant Physiol.

[20] Wang D, Ning K, Li J, Hu J, Han D, Wang H, Zeng X, Jing X, Zhou Q, Su X (2014). *Nannochloropsis* genomes reveal evolution of microalgal oleaginous traits. PLoS Genet.

[21] Li J, Han D, Wang D, Ning K, Jia J, Wei L, Jing X, Huang S, Chen J, Li Y (2014). Choreography of transcriptomes and lipidomes of *Nannochloropsis* reveals the mechanisms of oil synthesis in microalgae. Plant Cell.

[22] Xin Y, Lu YD, Lee YY, Wei L, Jia J, Wang QT, Wang DM, Bai F, Hu HH, Hu Q (2017). Producing designer oils in industrial microalgae by rational modulation of co-evolving type-2 diacylglycerol acyltransferases. Mol Plant.

[23] Poliner E, Farre EM, Benning C (2018). Advanced genetic tools enable synthetic biology in the oleaginous microalgae *Nannochloropsis* sp. Plant Cell Rep.

[24] Wang D, Lu Y, Huang H, Xu J (2012). Establishing oleaginous microalgae research models for consolidated bioprocessing of solar energy. Adv Biochem Eng Biotechnol.

[25] Wang QT, Lu YD, Xin Y, Wei L, Huang S, Xu J (2016). Genome editing of model oleaginous microalgae *Nannochloropsis* spp. by CRISPR/Cas9. Plant J.

[26] Xin Y, Shen C, She Y, Chen H, Wang C, Wei L, Yoon K, Han D, Hu Q, Xu J (2018). Biosynthesis of triacylglycerol molecules with a tailored PUFA profile in industrial microalgae. Mol Plant.

[27] Gee CW, Niyogi KK (2017). The carbonic anhydrase CAH1 is an essential component of the carbon-concentrating mechanism in *Nannochloropsis oceanica*. Proc Natl Acad Sci USA.

[28] Wei L, Shen C, El Hajjami M, You W, Wang Q, Zhang P, Ji Y, Hu H, Hu Q, Poetsch A (2019). Knockdown of carbonate anhydrase elevates *Nannochloropsis* productivity at high CO_2_ level. Metab Eng..

[29] Huertas IE, Colman B, Espie GS (2002). Mitochondrial-driven bicarbonate transport supports photosynthesis in a marine microalga. Plant Physiol.

[30] Huertas IE, Espie GS, Colman B (2002). The energy source for CO_2_ transport in the marine microalga *Nannochloris atomus*. Planta.

[31] Nakajima K, Tanaka A, Matsuda Y (2013). SLC4 family transporters in a marine diatom directly pump bicarbonate from seawater. Proc Natl Acad Sci USA.

[32] Shi J, Yi K, Liu Y, Xie L, Zhou Z, Chen Y, Hu Z, Zheng T, Liu R, Chen Y (2015). Phosphoenolpyruvate carboxylase in *Arabidopsis* leaves plays a crucial role in carbon and nitrogen metabolism. Plant Physiol.

[33] Dong HP, Williams E, Wang DZ, Xie ZX, Hsia RC, Jenck A, Halden R, Li J, Chen F, Place AR (2013). Responses of *Nannochloropsis oceanica* IMET1 to long-term nitrogen starvation and recovery. Plant Physiol.

[34] Park J, Khuu N, Howard AS, Mullen RT, Plaxton WC (2012). Bacterial- and plant-type phosphoenolpyruvate carboxylase isozymes from developing castor oil seeds interact in vivo and associate with the surface of mitochondria. Plant J.

[35] Mallona I, Egea-Cortines M, Weiss J (2011). Conserved and divergent rhythms of crassulacean acid metabolism-related and core clock gene expression in the cactus *Opuntia ficus*-*indica*. Plant Physiol.

[36] McKinlay JB, Shachar-Hill Y, Zeikus JG, Vieille C (2007). Determining *Actinobacillus succinogenes* metabolic pathways and fluxes by NMR and GC-MS analyses of 13C-labeled metabolic product isotopomers. Metab Eng.

[37] Zhang X, Jantama K, Moore JC, Jarboe LR, Shanmugam KT, Ingram LO (2009). Metabolic evolution of energy-conserving pathways for succinate production in *Escherichia coli*. Proc Natl Acad Sci USA.

[38] Fromm S, Braun HP, Peterhansel C (2016). Mitochondrial gamma carbonic anhydrases are required for complex I assembly and plant reproductive development. New Phytol.

[39] Soto D, Cordoba JP, Villarreal F, Bartoli C, Schmitz J, Maurino VG, Braun HP, Pagnussat GC, Zabaleta E (2015). Functional characterization of mutants affected in the carbonic anhydrase domain of the respiratory complex I in *Arabidopsis thaliana*. Plant J.

[40] Perales M, Parisi G, Fornasari M, Colaneri A, Villarreal F, Gonzalez-Schain N, Echave J, Gomez-Casati D, Braun HP, Araya A (2004). Gamma carbonic anhydrase like complex interact with plant mitochondrial complex I. Plant Mol Biol.

[41] Foyer CH, Bloom AJ, Queval G, Noctor G (2009). Photorespiratory metabolism: genes, mutants, energetics, and redox signaling. Annu Rev Plant Biol.

[42] Allen AE, Dupont CL, Obornik M, Horak A, Nunes-Nesi A, McCrow JP, Zheng H, Johnson DA, Hu H, Fernie AR (2011). Evolution and metabolic significance of the urea cycle in photosynthetic diatoms. Nature.

[43] Yamano T, Fukuzawa H (2009). Carbon-concentrating mechanism in a green alga, *Chlamydomonas reinhardtii*, revealed by transcriptome analyses. J Basic Microbiol.

[44] Merchant SS, Prochnik SE, Vallon O, Harris EH, Karpowicz SJ, Witman GB, Terry A, Salamov A, Fritz-Laylin LK, Marechal-Drouard L (2007). The *Chlamydomonas* genome reveals the evolution of key animal and plant functions. Science.

[45] Tsuji Y, Nakajima K, Matsuda Y (2017). Molecular aspects of the biophysical CO_2_-concentrating mechanism and its regulation in marine diatoms. J Exp Bot.

[46] Foyer CH, Neukermans J, Queval G, Noctor G, Harbinson J (2012). Photosynthetic control of electron transport and the regulation of gene expression. J Exp Bot.

[47] McGinn PJ, Morel FMM (2008). Expression and inhibition of the carboxylating and decarboxylating enzymes in the photosynthetic C4 pathway of marine diatoms. Plant Physiol.

[48] Fang W, Si Y, Douglass S, Casero D, Merchant SS, Pellegrini M, Ladunga I, Liu P, Spalding MH (2012). Transcriptome-wide changes in *Chlamydomonas reinhardtii* gene expression regulated by carbon dioxide and the CO_2_-concentrating mechanism regulator CIA5/CCM1. Plant Cell.

[49] Michel G, Tonon T, Scornet D, Cock JM, Kloareg B (2010). Central and storage carbon metabolism of the brown alga *Ectocarpus siliculosus*: insights into the origin and evolution of storage carbohydrates in Eukaryotes. New Phytol.

[50] Bauwe H, Hagemann M, Kern R, Timm S (2012). Photorespiration has a dual origin and manifold links to central metabolism. Curr Opin Plant Biol.

[51] Lin WR, Lai YC, Sung PK, Tan SI, Chang CH, Chen CY, Chang JS, Ng IS (2018). Enhancing carbon capture and lipid accumulation by genetic carbonic anhydrase in microalgae. J Taiwan Inst Chem E.

[52] Kang NK, Jeon S, Kwon S, Koh HG, Shin SE, Lee B, Choi GG, Yang JW, Jeong BR, Chang YK (2015). Effects of overexpression of a bHLH transcription factor on biomass and lipid production in *Nannochloropsis salina*. Biotechnol Biofuels.

[53] Baker NR (2008). Chlorophyll fluorescence: a probe of photosynthesis in vivo. Annu Rev Plant Biol.

[54] Lee TM, Tseng YF, Cheng CL, Chen YC, Lin CS, Su HY, Chow TJ, Chen CY, Chang JS (2017). Characterization of a heat-tolerant *Chlorella* sp. GD mutant with enhanced photosynthetic CO_2_ fixation efficiency and its implication as lactic acid. Biotechnol Biofuels.

[55] Lin Q, Gu N, Li G, Lin J, Huang L, Tan L (2012). Effects of inorganic carbon concentration on carbon formation, nitrate utilization, biomass and oil accumulation of *Nannochloropsis oculata* CS 179. Bioresour Technol.

[56] Jia J, Han DX, Gerken HG, Li YT, Sommerfeld M, Hu Q, Xu J (2015). Molecular mechanisms for photosynthetic carbon partitioning into storage neutral lipids in *Nannochloropsis oceanica* under nitrogen-depletion conditions. Algal Res.

[57] Lisec J, Schauer N, Kopka J, Willmitzer L, Fernie AR (2006). Gas chromatography mass spectrometry-based metabolite profiling in plants. Nat Protoc.

[58] Weckwerth W, Loureiro ME, Wenzel K, Fiehn O (2004). Differential metabolic networks unravel the effects of silent plant phenotypes. Proc Natl Acad Sci USA.

[59] Kaplan F, Kopka J, Haskell DW, Zhao W, Schiller KC, Gatzke N, Sung DY, Guy CL (2004). Exploring the temperature-stress metabolome of *Arabidopsis*. Plant Physiol.

[60] Trapnell C, Pachter L, Salzberg SL (2009). TopHat: discovering splice junctions with RNA-Seq. Bioinformatics.

[61] Trapnell C, Williams BA, Pertea G, Mortazavi A, Kwan G, van Baren MJ, Salzberg SL, Wold BJ, Pachter L (2010). Transcript assembly and quantification by RNA-Seq reveals unannotated transcripts and isoform switching during cell differentiation. Nat Biotechnol.

[62] Saeed AI, Bhagabati NK, Braisted JC, Liang W, Sharov V, Howe EA, Li J, Thiagarajan M, White JA, Quackenbush J (2006). TM4 microarray software suite. Methods Enzymol.

[63] Yeung KY, Haynor DR, Ruzzo WL (2001). Validating clustering for gene expression data. Bioinformatics.

[64] Guenin S, Mauriat M, Pelloux J, Van Wuytswinkel O, Bellini C, Gutierrez L (2009). Normalization of qRT-PCR data: the necessity of adopting a systematic, experimental conditions-specific, validation of references. J Exp Bot.

[65] Dyballa N, Metzger S (2009). Fast and sensitive colloidal coomassie G-250 staining for proteins in polyacrylamide gels. J Vis Exp.

[66] Schluesener D, Fischer F, Kruip J, Rogner M, Poetsch A (2005). Mapping the membrane proteome of *Corynebacterium glutamicum*. Proteomics.

[67] Cox J, Neuhauser N, Michalski A, Scheltema RA, Olsen JV, Mann M (2011). Andromeda: a peptide search engine integrated into the MaxQuant environment. J Proteome Res.

[68] Tyanova S, Temu T, Cox J (2016). The MaxQuant computational platform for mass spectrometry-based shotgun proteomics. Nat Protocols.

[69] Cox J, Mann M (2008). MaxQuant enables high peptide identification rates, individualized ppb-range mass accuracies and proteome-wide protein quantification. Nat Biotechnol.

[70] Cui YY, Wang ZR, Chen SW, Vainstein A, Ma HQ (2019). Proteome and transcriptome analyses reveal key molecular differences between quality parameters of commercial-ripe and tree-ripe fig (*Ficus carica* L). BMC Plant Biol.

[71] Maier T, Guell M, Serrano L (2009). Correlation of mRNA and protein in complex biological samples. FEBS Lett.

[72] Abraham PE, Yin HF, Borland AM, Weighill D, Lim SD, De Paoli HC, Engle N, Jones PC, Agh R, Weston DJ (2016). Transcript, protein and metabolite temporal dynamics in the CAM plant Agave. Nat Plants.

[73] Petersen TN, Brunak S, von Heijne G, Nielsen H (2011). SignalP 4.0: discriminating signal peptides from transmembrane regions. Nat Methods.

[74] Emanuelsson O, Nielsen H, von Heijne G (1999). ChloroP, a neural network-based method for predicting chloroplast transit peptides and their cleavage sites. Protein Sci.

[75] Claros MG (1995). MitoProt, a Macintosh application for studying mitochondrial proteins. Comput Appl Biosci.

[76] Gschloessl B, Guermeur Y, Cock JM (2008). HECTAR: a method to predict subcellular targeting in heterokonts. BMC Bioinform.

[77] Clement R, Dimnet L, Maberly SC, Gontero B (2016). The nature of the CO_2_-concentrating mechanisms in a marine diatom, *Thalassiosira pseudonana*. New Phytol.

[78] Aragon C, Pascual P, Gonzalez J, Escalona M, Carvalho L, Amancio S (2013). The physiology of ex vitro pineapple (*Ananas comosus* L. *Merr* var. MD-2) as CAM or C3 is regulated by the environmental conditions: proteomic and transcriptomic profiles. Plant Cell Rep.

