# Supplementary material for: Transcriptomic and proteomic responses to very low CO2 suggest multiple carbon concentrating mechanisms in Nannochloropsis oceanica
Source: Biotechnol Biofuels. 2019 Jun 28;12:168. doi: 10.1186/s13068-019-1506-8 (PMC6599299; doi:10.1186/s13068-019-1506-8)
Supplement: Supplementary file 1 — Additional file 1: Figure S1. A schematic diagram of the experimental design. Nannochloropsis oceanica cells were firstly grown to logarithmic phase under air enriched with 5% CO2. After adaption to new environment under 5% CO2 for an hour, the cells were then cultured under either 100 pm or 50,000 ppm CO2 concentration. Samples were collected at 0, 3, 6, 12 and 24 h from each condition (three biological replicate columns for each) by syringe for physiological characterization and multi-omics (transcriptome, proteome and metabolome) profiling. [file 13068_2019_1506_MOESM1_ESM.ppt]

## Slide 1
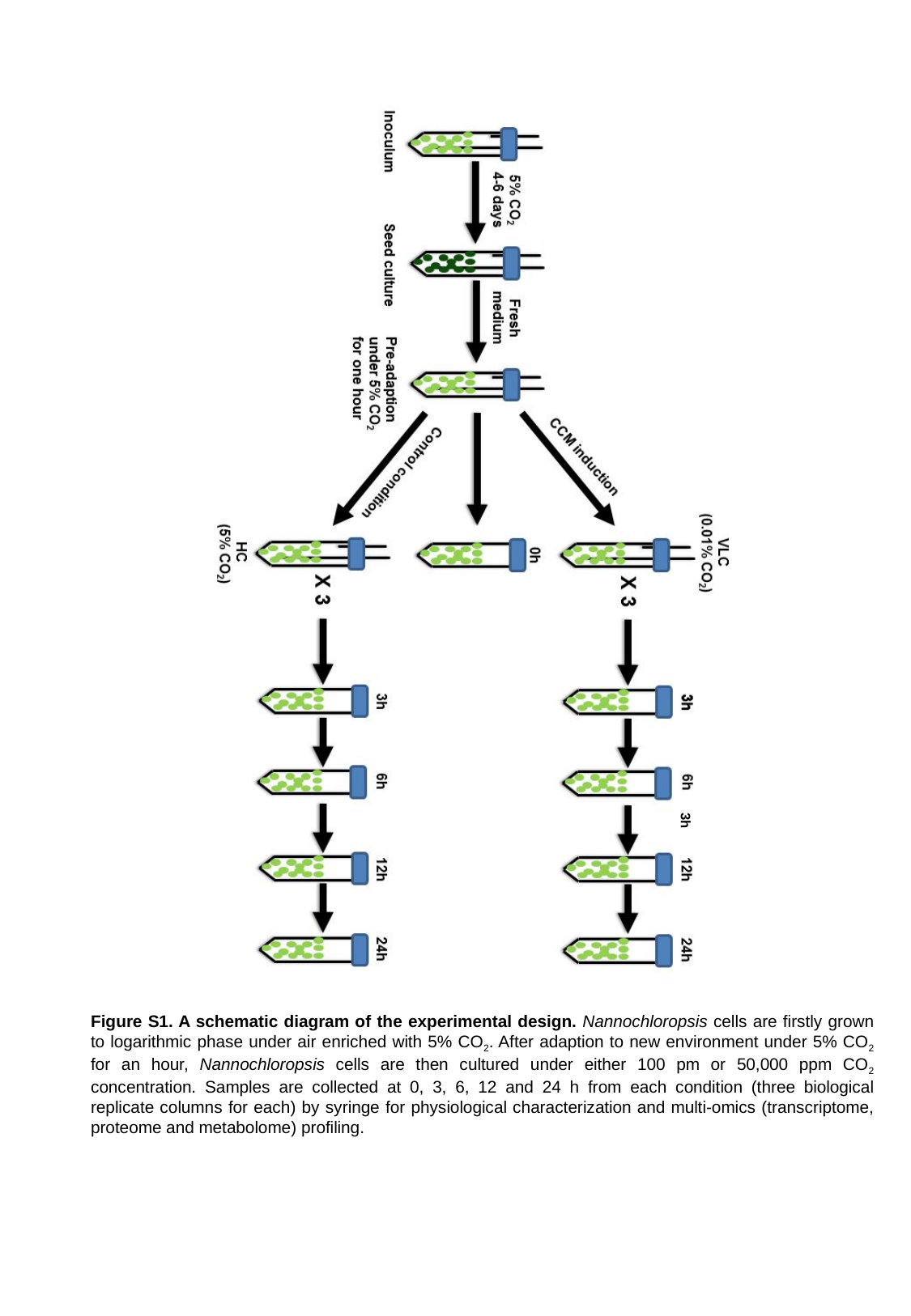

3h
Figure S1. A schematic diagram of the experimental design. Nannochloropsis cells are firstly grown to logarithmic phase under air enriched with 5% CO2. After adaption to new environment under 5% CO2 for an hour, Nannochloropsis cells are then cultured under either 100 pm or 50,000 ppm CO2 concentration. Samples are collected at 0, 3, 6, 12 and 24 h from each condition (three biological replicate columns for each) by syringe for physiological characterization and multi-omics (transcriptome, proteome and metabolome) profiling.
