# Supplementary material for: Transcriptomic and proteomic responses to very low CO2 suggest multiple carbon concentrating mechanisms in Nannochloropsis oceanica
Source: Biotechnol Biofuels. 2019 Jun 28;12:168. doi: 10.1186/s13068-019-1506-8 (PMC6599299; doi:10.1186/s13068-019-1506-8)
Supplement: Supplementary file 2 — Additional file 2: Figure S2. Growth curves and photosynthetic efficiency of N. oceanica IMET1 cells under VLC and HC conditions. (A) Growth curve of Nannochloropsis under two CO2 concentrations. The y-axis presents the average optical densities of triplicate algal cultures at 750 nm at each time point. Data are averages of at least three independent experiments (error bars represent standard deviations). (B) Maximum quantum efficiency (Fv/Fm) and active activity (Fv’/Fm’) of Photosystem II under VLC and HC conditions. The sharp decrease in Fv/Fm observed at 3h might be caused by short-term acclimation of the microalga to low carbon. (C) Change in concentration of total dissolved inorganic carbon (DIC) in the medium under VLC and HC conditions. The rapid increase in DIC for cells cultivated under HC was due to the start of aeration with 5% CO2. [file 13068_2019_1506_MOESM2_ESM.ppt]

## Slide 1
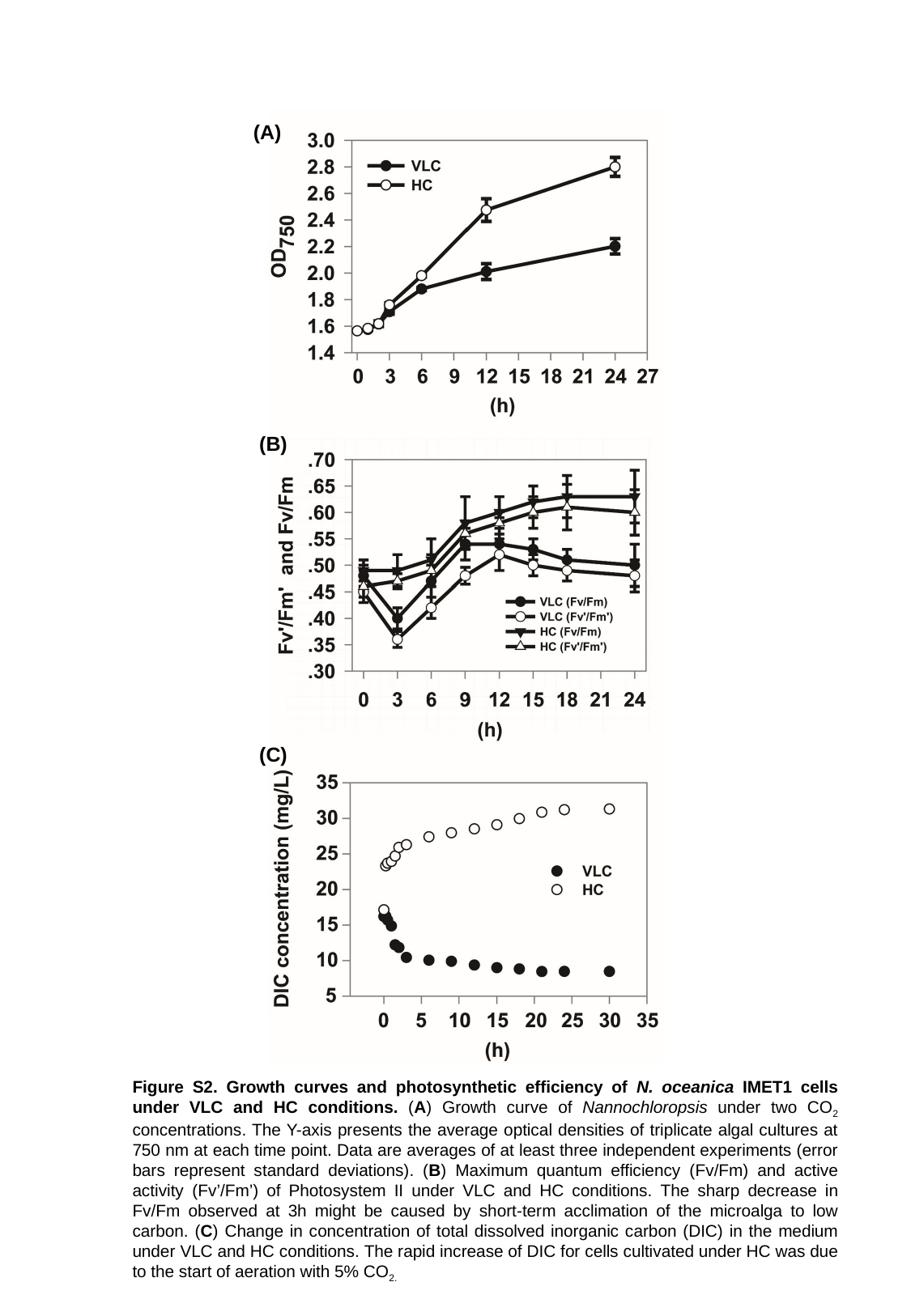

(A)
(B)
(C)
Figure S2. Growth curves and photosynthetic efficiency of N. oceanica IMET1 cells under VLC and HC conditions. (A) Growth curve of Nannochloropsis under two CO2 concentrations. The Y-axis presents the average optical densities of triplicate algal cultures at 750 nm at each time point. Data are averages of at least three independent experiments (error bars represent standard deviations). (B) Maximum quantum efficiency (Fv/Fm) and active activity (Fv’/Fm’) of Photosystem II under VLC and HC conditions. The sharp decrease in Fv/Fm observed at 3h might be caused by short-term acclimation of the microalga to low carbon. (C) Change in concentration of total dissolved inorganic carbon (DIC) in the medium under VLC and HC conditions. The rapid increase of DIC for cells cultivated under HC was due to the start of aeration with 5% CO2.
