# Supplementary material for: Transcriptomic and proteomic responses to very low CO2 suggest multiple carbon concentrating mechanisms in Nannochloropsis oceanica
Source: Biotechnol Biofuels. 2019 Jun 28;12:168. doi: 10.1186/s13068-019-1506-8 (PMC6599299; doi:10.1186/s13068-019-1506-8)
Supplement: Supplementary file 3 — Additional file 3: Table S1. General information on the time series mRNA-Seq and proteome datasets under VLC and HC. [file 13068_2019_1506_MOESM3_ESM.doc]

**Table S1. General information on the time-series mRNA-Seq and proteome datasets under VLC and HC.**

| **Transcriptome** | | | | | | | | |
| --- | --- | --- | --- | --- | --- | --- | --- | --- |
| **Sample** | **Clean PE reads** | | **Clean reads** | | **Uniquely aligned reads** | | **Aligned ratio** | **Aligned genes** |
| **0h-1** | 12,668,038 | | 11,959,924 | | 10,716,856 | | 84.6% | 9,445 |
| **0h-2** | 14,958,548 | | 13,970,720 | | 12,555,586 | | 83.9% | 9,423 |
| **HC-3h-1** | 17,351,522 | | 15,568,864 | | 14,649,035 | | 84.4% | 9,476 |
| **HC-3h-2** | 14,091,410 | | 12,789,796 | | 12,341,979 | | 87.6% | 9,416 |
| **HC-6h-1** | 24,349,768 | | 22,582,302 | | 21,172,785 | | 87.0% | 9,523 |
| **HC-6h-2** | 24,521,288 | | 23,619,066 | | 21,287,251 | | 86.8% | 9,512 |
| **HC-12h-1** | 16,619,496 | | 15,568,864 | | 14,197,728 | | 85.4% | 9,469 |
| **HC-12h-2** | 11,832,804 | | 10,089,796 | | 10,111,255 | | 85.5% | 9,472 |
| **HC-24h-1** | 25,203,098 | | 22,816,042 | | 21,721,119 | | 86.2% | 9,503 |
| **HC-24h-2** | 20,599,364 | | 18,837,258 | | 17,784,950 | | 86.3% | 9,543 |
| **VLC-3h-1** | 14,588,712 | | 16,665,614 | | 12,626,747 | | 86.6% | 9,462 |
| **VLC-3h-2** | 16,923,136 | | 15,643,056 | | 14,820,733 | | 87.6% | 9,479 |
| **VLC-6h-1** | 28,904,566 | | 26,754,704 | | 24,987,938 | | 86.4% | 9,542 |
| **VLC-6h-2** | 19,421,254 | | 17,060,678 | | 16,813,773 | | 86.6% | 9,527 |
| **VLC-12h-1** | 14,357,158 | | 18,259,242 | | 12,179,367 | | 84.8% | 9,592 |
| **VLC-12h-2** | 16,605,438 | | 15,899,204 | | 14,134,439 | | 85.1% | 9,593 |
| **VLC-24h-1** | 23,981,358 | | 21,331,514 | | 20,613,339 | | 86.0% | 9,560 |
| **VLC-24h-2** | 24,648,986 | | 22,906,404 | | 21,174,281 | | 85.9% | 9,557 |
| **Proteome** | | | | | | | | |
| **Sample** | | **PSM** | | **Total proteins** | | **Proteins after filtering** | | |
| **0h-1** | | 22,901 | | 1,953 | | 1,732 | | |
| **0h-2** | | 23,586 | | 1,991 | | 17,58 | | |
| **0h-3** | | 24,344 | | 2,003 | | 1,751 | | |
| **HC-3h-1** | | 23,278 | | 1,828 | | 1,687 | | |
| **HC-3h-2** | | 24,226 | | 1,909 | | 1,744 | | |
| **HC-3h-3** | | 15,763 | | 1,425 | | 1,356 | | |
| **HC-6h-1** | | 29,548 | | 2,103 | | 1,808 | | |
| **HC-6h-2** | | 23,264 | | 1,882 | | 1,719 | | |
| **HC-6h-3** | | 25,832 | | 1,955 | | 1,747 | | |
| **HC-12h 1** | | 24,607 | | 1,627 | | 1,496 | | |
| **HC-12h-2** | | 25,579 | | 1,639 | | 1,505 | | |
| **HC-12h-3** | | 26,529 | | 1,709 | | 1,585 | | |
| **HC-24h-1** | | 30,115 | | 1,711 | | 1,581 | | |
| **HC-24h-2** | | 29,277 | | 1,629 | | 1,512 | | |
| **HC-24h-3** | | 28,071 | | 1,516 | | 1,432 | | |
| **VLC-3h-1** | | 22,138 | | 1,841 | | 1,685 | | |
| **VLC-3h-2** | | 23,725 | | 1,868 | | 1,689 | | |
| **VLC-3h-3** | | 24,289 | | 1,890 | | 1,705 | | |
| **VLC-6h-1** | | 23,637 | | 1,754 | | 1,629 | | |
| **VLC-6h-2** | | 25,634 | | 1,873 | | 1,687 | | |
| **VLC-6h-3** | | 22,174 | | 1,789 | | 1,667 | | |
| **VLC-12h-1** | | 27,471 | | 1,994 | | 1,739 | | |
| **VLC-12h-2** | | 25,910 | | 1,951 | | 1,695 | | |
| **VLC-12h-3** | | 27,255 | | 1,829 | | 1,640 | | |
| **VLC-24h-1** | | 26,594 | | 1,652 | | 1,540 | | |
| **VLC-24h-2** | | 20,449 | | 1,349 | | 1,289 | | |
| **VLC-24h-3** | | 27,279 | | 1,609 | | 1,482 | | |

PE: Pair end; PSM: Peptide-spectrum match.
