# Supplementary material for: Transcriptomic and proteomic responses to very low CO2 suggest multiple carbon concentrating mechanisms in Nannochloropsis oceanica
Source: Biotechnol Biofuels. 2019 Jun 28;12:168. doi: 10.1186/s13068-019-1506-8 (PMC6599299; doi:10.1186/s13068-019-1506-8)
Supplement: Supplementary file 5 — Additional file 5: Table S3. Real-time PCR primer sequences for the 12 genes used in real-time PCR experiments to validate the mRNA-Seq results. [file 13068_2019_1506_MOESM5_ESM.doc]

**Table S3. Real-time PCR primer sequences for the 12 genes used in real-time PCR experiments to validate the mRNA-Seq results.**

| **Gene name** | **Gene ID** | **Forward primer** | **Reverse primer** |
| --- | --- | --- | --- |
| **Actin** | s00082.g3056 | 5'GCCGTTATTGGATGGATATG3' | 5'ACAACAACTCTCCTTCACA3' |
| **γ-CA1** | s00026.g1084 | 5'ATCACAGTCGGAGAACAA3' | 5'CAGCCTTATCCAGAACCT3' |
| **β-CA2** | s00043.g2018 | 5' CTGGAAAGGAAAGGAGAGA3' | 5' AGAGTCGGAACAAGCAATA3' |
| **β-CA3** | s00066.g2209 | 5' ACCAAGCATCATCATCCT3' | 5' GTCGTAGAGTAGCCTGTG3' |
| **β-CA4** | s00140.g4812 | 5'TTATTTGAAATGTGGTGTATTGC3' | 5'CTGACAGAAGAGACTATGGT3' |
| **α-CA5** | s00214.g6125 | 5' ACCTTGAGAAGATGGAGTC3' | 5' CCGACAGTCTGGAAGTTA3' |
| **BCT1** | s00007.g19 | 5' CCGATACATTCACAACTTCTC3' | 5' CACCAACCTCACCGTAAT3' |
| **BCT2** | s00043.g1855 | 5' TCCTCCTATTCCTCCTCAT3' | 5' AACCATTCTATTCTCCATCCT3' |
| **PEPC** | s00145.g5140 | 5' TGGATTGCTTGGAGACTT3' | 5' TCTTGCTTGCCTTGGATA3' |
| **PEPCK** | s00247.g6884 | 5' CCTTGTTCTTCGGTCTCT3' | 5' TCTTGCTCGTGTTGTAGT3' |
| **PPDK1** | s00095.g3407 | 5' GATGACCACGGAGAAGAT3' | 5' GGAGAGCACGAGACATAA3' |
| **SHMT** | s00043.g1924 | 5'AAGGCTCTGACTGCTAAG3' | 5' CTTGAATCCACGACTTGTC3' |
| **GGAT** | s00096.g3637 | 5' ATGGTCGGCTACTACTTG3' | 5' CACGCTCTGATAGATATTGTC3' |
