# Supplementary material for: Transcriptomic and proteomic responses to very low CO2 suggest multiple carbon concentrating mechanisms in Nannochloropsis oceanica
Source: Biotechnol Biofuels. 2019 Jun 28;12:168. doi: 10.1186/s13068-019-1506-8 (PMC6599299; doi:10.1186/s13068-019-1506-8)
Supplement: Supplementary file 6 — Additional file 6: Figure S3. Validation of mRNA-Seq-based transcript quantification using real-time quantitative PCR (qPCR). Twelve genes involved in CCM and photorespiration metabolism are selected for qPCR validation. The genes and qPCR primer sequences are listed in Additional file 5: Table S3. The transcript levels at each time point after the onset of carbon limitation are included, and the correlation coefficient between the average qPCR-based transcript abundance and the mRNA-Seq-based transcript abundance is 0.9497 (R2). [file 13068_2019_1506_MOESM6_ESM.ppt]

## Slide 1
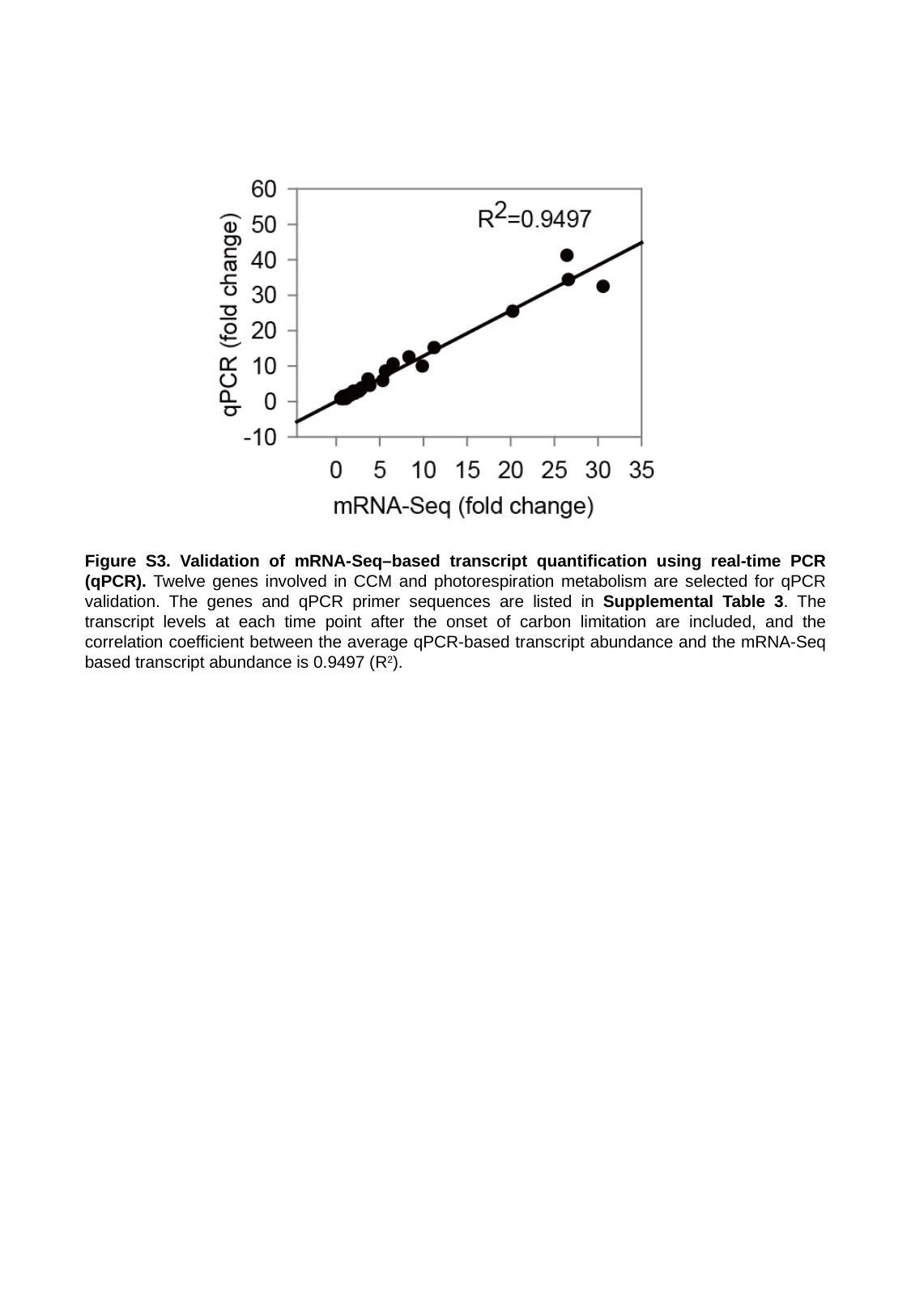

Figure S3. Validation of mRNA-Seq–based transcript quantification using real-time PCR (qPCR). Twelve genes involved in CCM and photorespiration metabolism are selected for qPCR validation. The genes and qPCR primer sequences are listed in Supplemental Table 3. The transcript levels at each time point after the onset of carbon limitation are included, and the correlation coefficient between the average qPCR-based transcript abundance and the mRNA-Seq based transcript abundance is 0.9497 (R2).
