# Supplementary material for: Transcriptomic and proteomic responses to very low CO2 suggest multiple carbon concentrating mechanisms in Nannochloropsis oceanica
Source: Biotechnol Biofuels. 2019 Jun 28;12:168. doi: 10.1186/s13068-019-1506-8 (PMC6599299; doi:10.1186/s13068-019-1506-8)
Supplement: Supplementary file 7 — Additional file 7: Figure S4. Transcript dynamics of the genes related to CCM and photorespiration metabolism in N. oceanica IMET1 as measured by mRNA-Seq and real-time quantitative PCR. Each data point represents the average of three biological replicates. Each sample was analyzed in technical triplicates. [file 13068_2019_1506_MOESM7_ESM.ppt]

## Slide 1
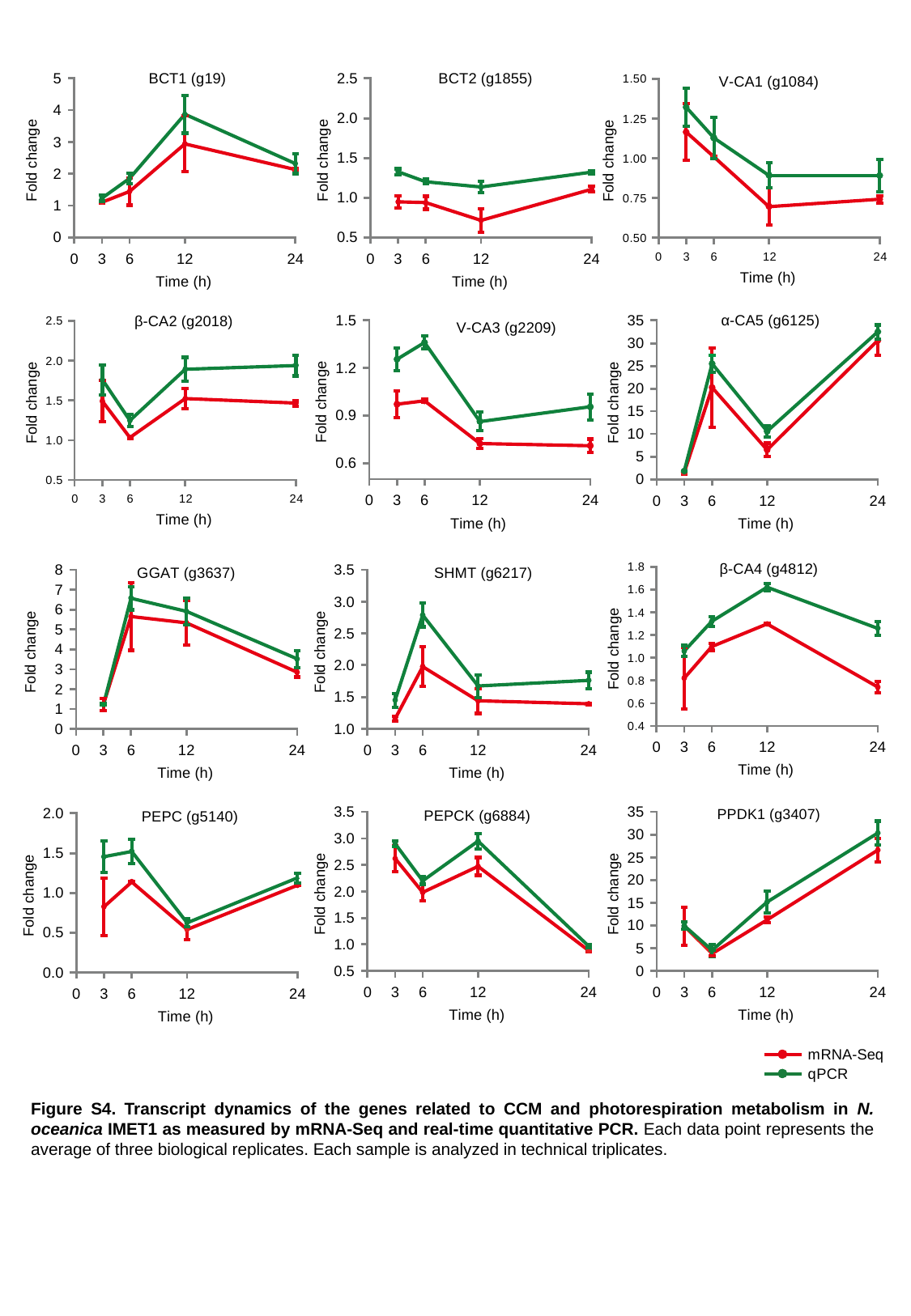

Figure S4. Transcript dynamics of the genes related to CCM and photorespiration metabolism in N. oceanica IMET1 as measured by mRNA-Seq and real-time quantitative PCR. Each data point represents the average of three biological replicates. Each sample is analyzed in technical triplicates.
