# Supplementary material for: Transcriptomic and proteomic responses to very low CO2 suggest multiple carbon concentrating mechanisms in Nannochloropsis oceanica
Source: Biotechnol Biofuels. 2019 Jun 28;12:168. doi: 10.1186/s13068-019-1506-8 (PMC6599299; doi:10.1186/s13068-019-1506-8)
Supplement: Supplementary file 11 — Additional file 11: Figure S5. Contents of carbohydrates, proteins and lipids under VLC and HC. The biochemical compositions in the microalga biomass were measured under VLC and HC. Data are shown as means ± SD of three replicates. [file 13068_2019_1506_MOESM11_ESM.pptx]

## Slide 1
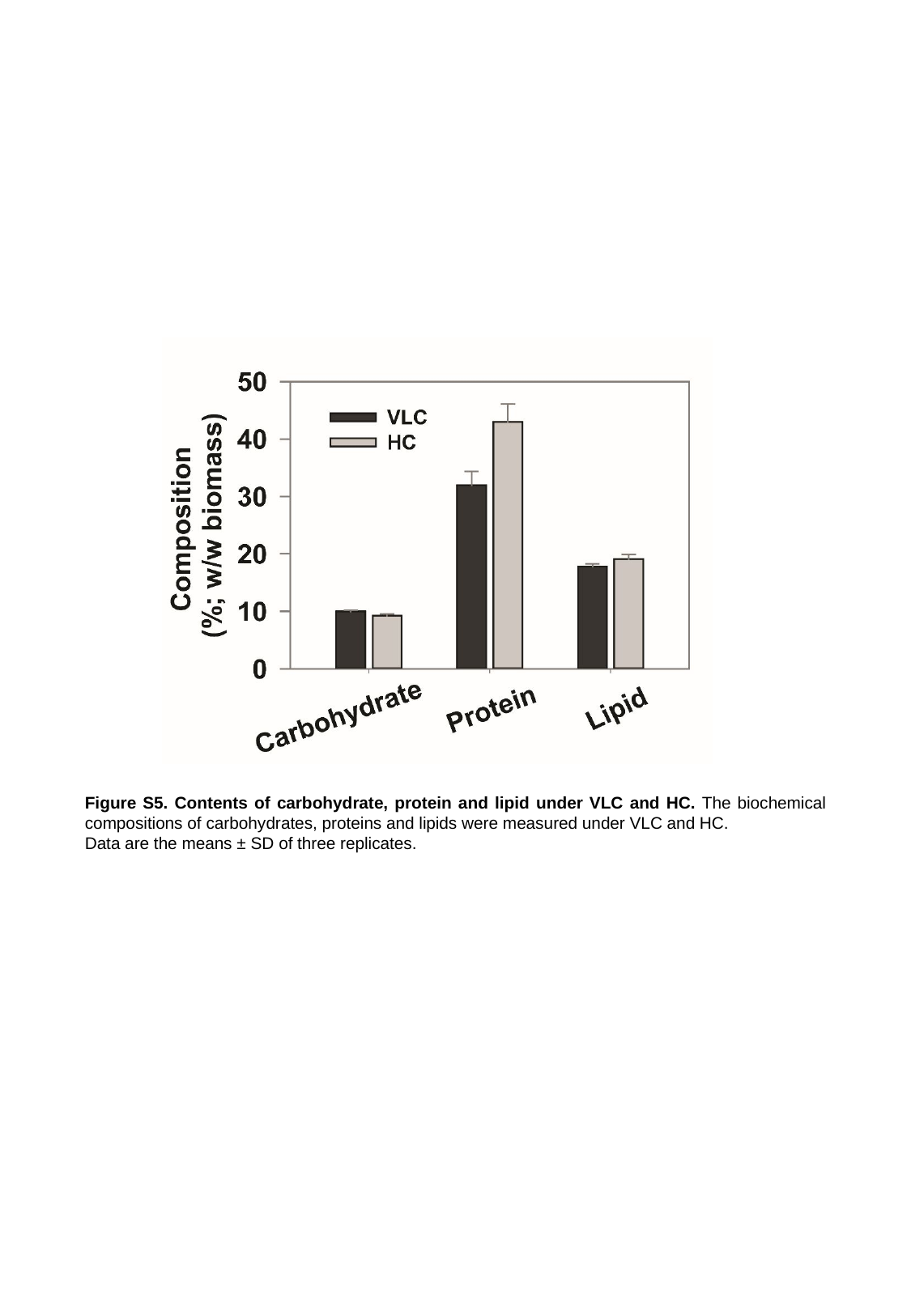

Figure S5. Contents of carbohydrate, protein and lipid under VLC and HC. The biochemical compositions of carbohydrates, proteins and lipids were measured under VLC and HC.
Data are the means ± SD of three replicates.
