# Supplementary material for: Transcriptomic and proteomic responses to very low CO2 suggest multiple carbon concentrating mechanisms in Nannochloropsis oceanica
Source: Biotechnol Biofuels. 2019 Jun 28;12:168. doi: 10.1186/s13068-019-1506-8 (PMC6599299; doi:10.1186/s13068-019-1506-8)
Supplement: Supplementary file 13 — Additional file 13: Figure S6. Proposed response of central carbon metabolism during CCM induction in N. oceanica IMET1. 1,3PG, 1,3-biphosphoglycerate; 2OG, α-ketoglutarate; 2PG, 2-phosphoglycerate; 3PG, 3-phosphoglycerate; AAT, ADP/ATP transporter; ACS, acetyl-CoA synthetase; ACT, aconitate; AHD, aconitate hydratase; ALDO, fructose-1,6-bisphosphate aldolase; CS, ATP citrate synthase; DHAP, dihydroxyacetone phosphate; ENL, enolase; ENR, enoyl-ACP reductase; F1,6P, fructose 1,6-biphosphate; FBP, fructose bisphosphatase; F6P, fructose-6-phosphate; FHD, fumarate hydratase; FUM, fumarate; G6P, glucose-6-phosphate; GK, glucose kinase; Glu, glucose; GPDH, glyceraldehyde-3-phosphate dehydrogenase; GPI, glucose phosphate isomerase; GS, 1,3-β-glucan synthase; HAD, hydroxyacyl-ACP dehydrogenase; ICDH, isocitrate dehydrogenase; ICIT, isocitrate; MHD, malate dehydrogenase; NTT, nucleotide transporter; OGDH, 2-oxoglutarate dehydrogenase; PGAM, phosphoglycerate mutase; PGK, phosphoglycerate kinase; PFK, phosphofructose kinase; PPDK, phosphate dikinase; PPT, Pi/PEP translocator; PYR, pyruvate; SCS, succinyl-CoA synthetase; SDH, succinate dehydrogenase; SUC, succinyl-CoA; TPI, triosephosphate isomerase; SFC, succinate/fumarate carrier; FBPA: fructose-1,6-bisphosphate aldolase; TL: transketolase; SBP: sedoheptulose bisphosphatase; RPI: ribose-5-phosphate isomerase; RPE: ribulose-phosphate 3-epimerase. The metabolites up- or downregulated based on the metabolomics analysis are indicated by red arrows. [file 13068_2019_1506_MOESM13_ESM.ppt]

## Slide 1
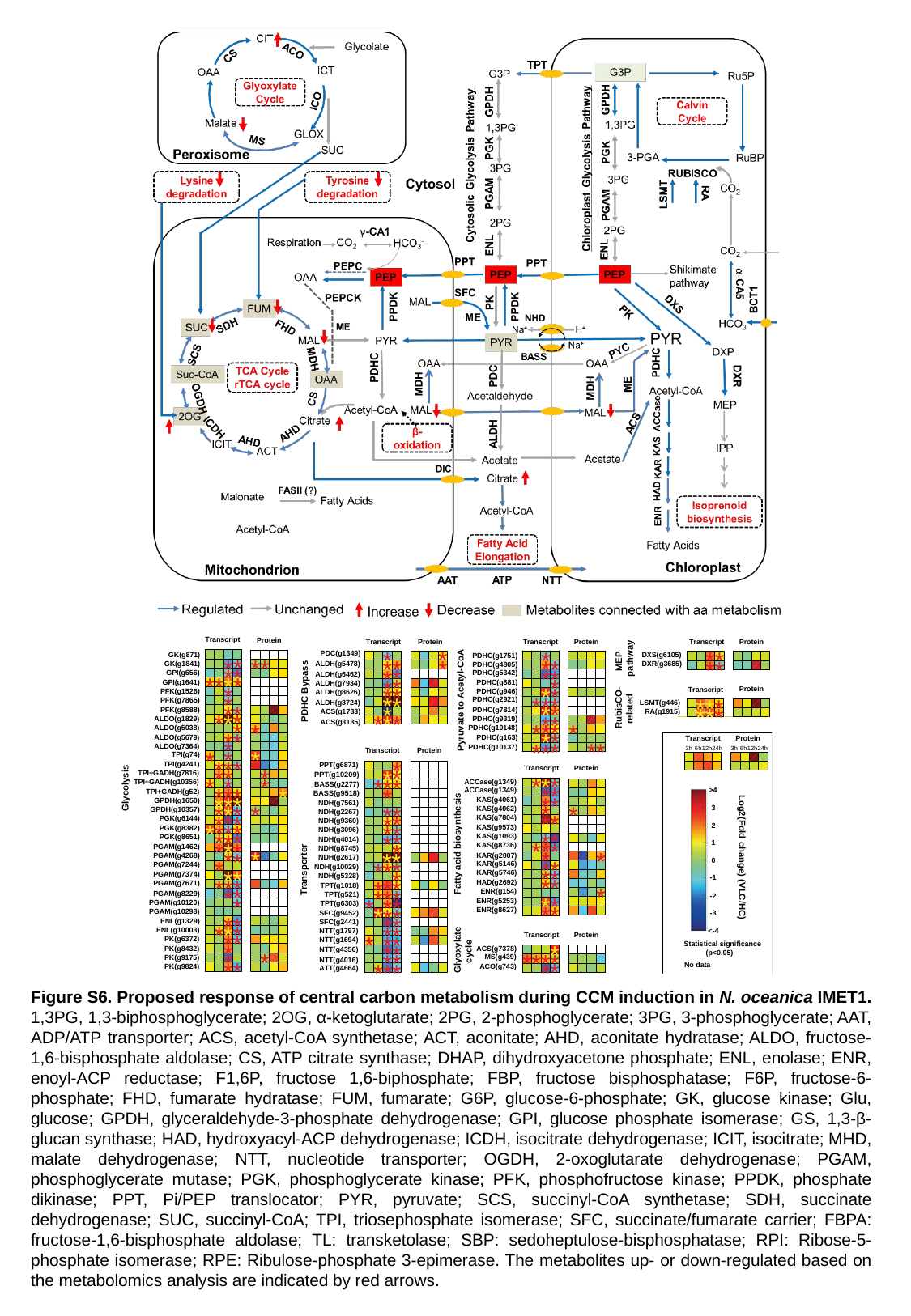

Figure S6. Proposed response of central carbon metabolism during CCM induction in N. oceanica IMET1. 1,3PG, 1,3-biphosphoglycerate; 2OG, α-ketoglutarate; 2PG, 2-phosphoglycerate; 3PG, 3-phosphoglycerate; AAT, ADP/ATP transporter; ACS, acetyl-CoA synthetase; ACT, aconitate; AHD, aconitate hydratase; ALDO, fructose-1,6-bisphosphate aldolase; CS, ATP citrate synthase; DHAP, dihydroxyacetone phosphate; ENL, enolase; ENR, enoyl-ACP reductase; F1,6P, fructose 1,6-biphosphate; FBP, fructose bisphosphatase; F6P, fructose-6-phosphate; FHD, fumarate hydratase; FUM, fumarate; G6P, glucose-6-phosphate; GK, glucose kinase; Glu, glucose; GPDH, glyceraldehyde-3-phosphate dehydrogenase; GPI, glucose phosphate isomerase; GS, 1,3-β-glucan synthase; HAD, hydroxyacyl-ACP dehydrogenase; ICDH, isocitrate dehydrogenase; ICIT, isocitrate; MHD, malate dehydrogenase; NTT, nucleotide transporter; OGDH, 2-oxoglutarate dehydrogenase; PGAM, phosphoglycerate mutase; PGK, phosphoglycerate kinase; PFK, phosphofructose kinase; PPDK, phosphate dikinase; PPT, Pi/PEP translocator; PYR, pyruvate; SCS, succinyl-CoA synthetase; SDH, succinate dehydrogenase; SUC, succinyl-CoA; TPI, triosephosphate isomerase; SFC, succinate/fumarate carrier; FBPA: fructose-1,6-bisphosphate aldolase; TL: transketolase; SBP: sedoheptulose-bisphosphatase; RPI: Ribose-5-phosphate isomerase; RPE: Ribulose-phosphate 3-epimerase. The metabolites up- or down-regulated based on the metabolomics analysis are indicated by red arrows.
